# SHARE-Topic: Bayesian interpretable modeling of single-cell multi-omic data

**DOI:** 10.1186/s13059-024-03180-3

**Published:** 2024-02-23

**Authors:** Nour El Kazwini, Guido Sanguinetti

**Affiliations:** Theoretical and Scientific Data Science, Scuola Internazionale Superiore di Studi Avanzati, Trieste, Italy

**Keywords:** Gene regulation, Single-cell multi-omics, Bayesian modeling, Interpretability, Gene regulator in cancer, Lymphoma

## Abstract

Multi-omic single-cell technologies, which simultaneously measure the transcriptional and epigenomic state of the same cell, enable understanding epigenetic mechanisms of gene regulation. However, noisy and sparse data pose fundamental statistical challenges to extract biological knowledge from complex datasets. SHARE-Topic, a Bayesian generative model of multi-omic single cell data using topic models, aims to address these challenges. SHARE-Topic identifies common patterns of co-variation between different omic layers, providing interpretable explanations for the data complexity. Tested on data from different technological platforms, SHARE-Topic provides low dimensional representations recapitulating known biology and defines associations between genes and distal regulators in individual cells.

**Supplementary Information:**

The online version contains supplementary material available at 10.1186/s13059-024-03180-3.

## Background

Biological complexity arises from interactions of many molecular factors at varying spatial and temporal scales. Understanding the nature and dynamics of these interactions is a major open problem in fundamental biology, with potentially important translational implications. Over the last two decades, the emergence of next-generation sequencing technologies, and more recently of single-cell sequencing technologies, has been a major accelerator towards tackling these questions, with large international consortia such as ENCODE and the Human Cell Atlas [1, 2] providing the community with invaluable data sets measuring a variety of molecular features potentially influencing gene expression.

Recent breakthroughs in single-cell technology have opened the possibility of measuring, in a high-throughput fashion, multiple molecular layers within the same cell, providing new opportunities to enhance our understanding of the interactions between biological factors. These technologies, collectively referred to as single-cell (sc) multi-omics, are generally designed to measure simultaneously the cell’s transcriptome together with one or more other molecular features, typically epigenetic factors such as DNA methylation or chromatin accessibility, DNA sequence or presence of protein markers [3–8]. These sc multi-omics technologies offer in principle a number of enticing possibilities, chief among them the opportunity to measure gene regulatory mechanisms in individual cells, and its variability across cells.

In practice, analyzing and interpreting sc multi-omic data present considerable challenges, due to the high level of sparsity and noisiness of the data [9]. To date, some of the most effective strategies adapt methods developed in different contexts to sc multiomics, such as MOFA+ [10], an extension of the multi-omic factor analysis method devised for bulk multiomics [11], or the Seurat/Signac suite [12, 13], originally devised to integrate multiple ’omics layers from different cells. These methods rely generally on linear dimensionality reduction, often prefaced by non-trivial pre-processing techniques. Alternatively, many recent efforts have focused on deploying deep learning methodologies within the autoencoder (AE) paradigm [14–18], sometimes integrated in non-trivial architectures such as graph neural networks [19]. AEs use non-linear maps (parametrised by deep neural networks) to explain the variability in the data in terms of a latent space, where each cell is represented by a low dimensional vector (typically of around ten dimensions, instead of tens of thousands of molecular features). Such nonlinear methods have been shown to outperform linear dimensionality reduction methods when used for tasks such as clustering cells and for survival analysis [14–16]. Despite these successes in extracting patterns at the cell level, obtaining insights at the gene level from AEs (for example in terms of specific regulatory interactions) is extremely difficult, due to the effective impossibility of reliably interpret the contribution of individual genes in complex nonlinear models. Indeed, even simple linear analyses, such as measuring correlations between region accessibility and gene expression, are very challenging in the single-cell realm due to the high levels of noise, as demonstrated recently in [20].

Here, we introduce SHARE-Topic, a Bayesian statistical model of joint chromatin accessibility and transcriptomic data, perhaps the most widely available type of sc multi-omic data. SHARE-Topic extends the cisTopic model of single-cell chromatin accessibility [21] by coupling the epigenomic state with gene expression through latent variables (topics) which are associated to regions and genes within an individual cell. In this way, SHARE-Topic is able to extract a latent space representation of each cell informed by both the epigenome and the transcriptome, but crucially also to model the joint variability of individual genes regions, providing an interpretable analysis tool which can help in generating novel hypotheses from the data. We test SHARE-Topic on five different data sets generated using three different single-cell multi-omics platforms: SHARE-seq, SNARE-seq [3] and the commercial 10X multi-ome platform. The performance demonstrates good scalability of the algorithm as well as its ability to extract novel biological information from these complex data sets. We show that SHARE-Topic is able to achieve competitive results in terms of dimensionality results against state of the art methods, and that it can effectively capture interactions between genes and regulatory regions.

**Table 1:** Interpolation of topic model to biological framework

Topic model	SHARE-Topic	Symbol
Documents	Cells	c
Words	Regions/genes reads	$r^c/n_g^c$
Topics: science, sports, music,...	Biological processes (cell differentiation, chemo-taxis...)	t
Topic-contribution to a document	Topic-contribution to a cell	$\theta _c^t$
Likelihood to find a word in a topic	Likelihood of: an open region/number of reads in a topic	$\phi _r^t/$ Poi$(\lambda _g^t)$

**Fig. 1:**
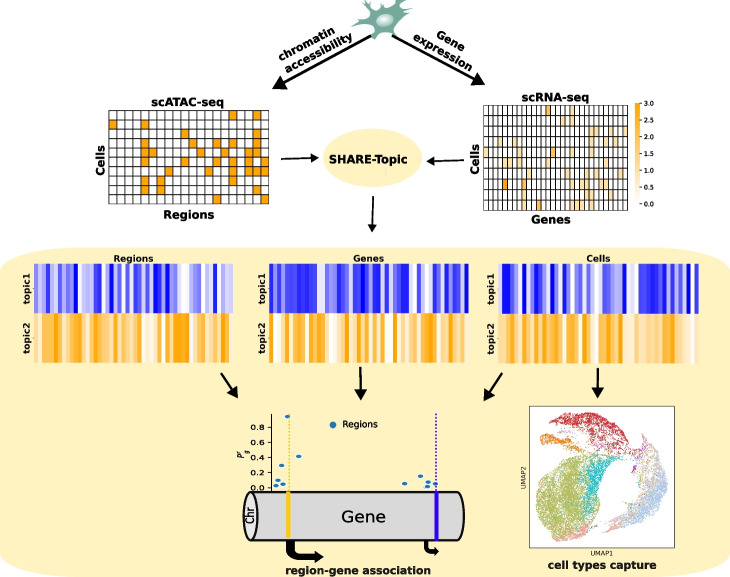
Workflow of SHARE-Topic: the scATAC-seq binary data and the expression matrix of the scRNA-seq data are fed to SHARE-Topic. SHARE-Topic extracts latent representation in topic space for each cell, gene, and region in the data. The latent representation of the cells is used to visualize the heterogeneity in cell types using Umap. The latent representations of genes and regions are used to extract biological interactions between genes and regions that shape the regulatory mechanisms in the cells

**Fig. 2:**
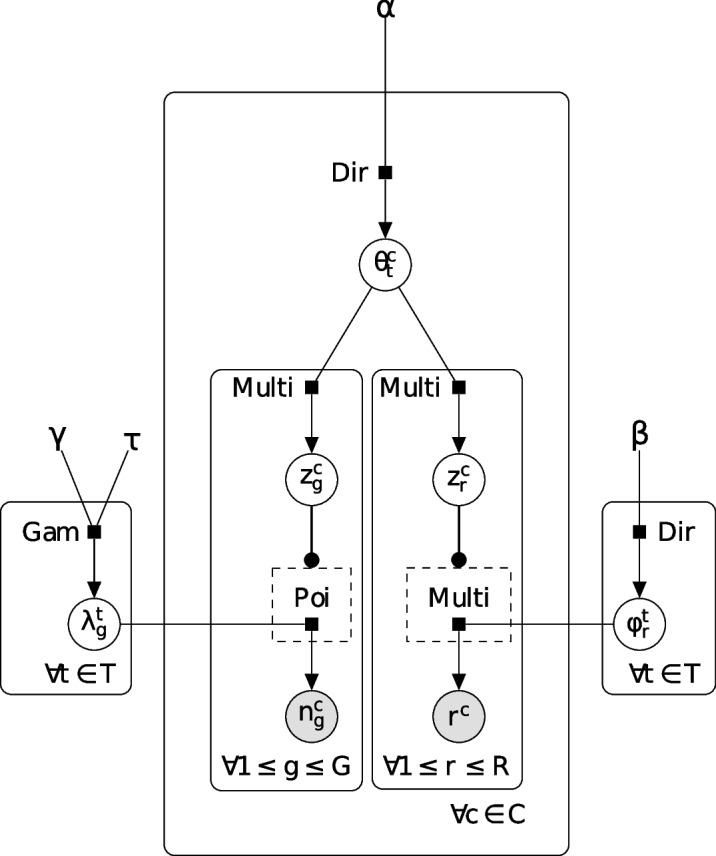
The graphical model of SHARE-Topic: graphical representation of the SHARE-Topic model illustrating the interrelationships between latent topics and observed gene expression reads ($n_g^c$) and chromatin regions observed ($r^c$). The model depicts the interactions on a given cell c between its transcriptomic profile and accessible chromatin region profile. These observations, according to SHARE-Topic are generated in the following way: each cell *c* is a different mixture of topics ($\theta ^c_t$). Given a contribution of a certain topic *t*, there is a likelihood to observe a gene count in the cell $n_g^c$ sampled from a Poisson distribution with an expected number of reads $\lambda _g^t$. On the other side also for a given topic *t *contribution in a cell, the likelihood of finding a region $r^c$ open is $\phi _r^t$. The priors are shown in the model at the top layer and descend down in a hierarchical fashion to observations

## Results

### The SHARE-Topic model

Topic models are unsupervised learning algorithms, originally designed to analyze and annotate large archives of text documents with thematic information [22–26]. The premise of topic modeling is that each document can be represented as a point in a much lower dimensional space (topic space), corresponding to the relative importance of different topics to the document. The probability of a word appearing in a document depends strongly on the topic, hence each topic is associated with a distinct distribution over word frequencies, which can also be used to associate topics with semantically meaningful annotations.

Bravo González-Blas et al. [21] have recently proposed cis-Topic, a topic model designed to efficiently analyze single-cell ATAC-seq data. cis-Topic provides an effective tool to obtain lower dimensional representations of the very high-dimensional scATAC-seq data, however interpretation of its latent space is complicated by the varying quality of the annotation of open chromatin regions. In this paper, we present SHARE-Topic, a topic model adapted to multi-omics data which allows both a stronger interpretability and gene-level predictions. A high-level view of the model structure is given in Fig. 1: single-cell multi-omics, encoded as two high-dimensional sparse matrices, is the input to SHARE-Topic. The model then utilizes a Gibbs sampler to obtain posterior estimates of the various parameters, which can be used both to obtain a low dimensional representation of the cells, and to associate topics to cells and regions to genes. The structure of the model is given in Fig. 2. A table illustrating the correspondence of concepts in classical (text based) topic modeling and their multi-omics analog in SHARE-Topic is given in Table 1.

### Data sets used

The field of single-cell multi-omics technology is still in rapid development, with multiple protocols being proposed and one already available commercially (the 10X multiome platform). To evaluate our results in a platform agnostic fashion, we sample extensively the space of sc multi-omics technologies. We use two data sets generated with the SHARE-seq technology [6], two data sets generated with the commercial 10X multiome platform, and one data set generated with the SNARE-seq technology. The SHARE-seq data sets profile approximately 3000 mouse brain cells and approximately 30,000 mouse skin cells; the number of genes/regions retained for each data set after pre-processing is of approximately 6000 genes expressed, and $2 \times 10^6$ regions for the brain data set, and approximately 3100 genes, and $9 \times 10^5$ regions for the skin data set. The multiome data sets profiles approximately 14,000 lymphoma cancer cells and 10,000 peripheral mononuclear blood cells (PMBC10k), retaining approximately 8000 genes and 9$\times 10^4$ regions for both. The SNARE-seq data set retained approximately 9000 cells, 5000 genes, and $5\times 10^4$ regions. Pre-processed data was obtained directly from the websites associated with the original data sets (see the “Availability of data and materials” section); in particular, chromatin accessibility was already provided as a binary matrix resulting from a peak-calling procedure. Details of the filtering procedure can be found in the “Methods” section.

### SHARE-Topic recapitulates cell identities

As with any other dimensionality reduction tool, from PCA to variational auto-encoders, a primary output of SHARE-Topic is the assignment of a latent vector to every cell. In our case, this vector is a probability distribution over the topics indicating to which topic each cell partakes. The choice of the number of topics (dimensionality of the latent space) is a non-trivial hyperparameter tuning issue; we resort to using the Widely Applicable Information Criterion (WAIC) [27] (more details on the criteria for the choice of topics number are given in the “Methods” section). The latent space can then be visualized using tools such as Uniform Manifold Approximation and Projection (UMAP) [28], and the consistency of the visualization with existing annotations can be assessed using quantitative criteria. While the main purpose of SHARE-Topic is to leverage the latent representation to understand biological interactions, it is still a useful quantitative benchmark to assess its capability of recapitulating cell identities.

**Fig. 3:**
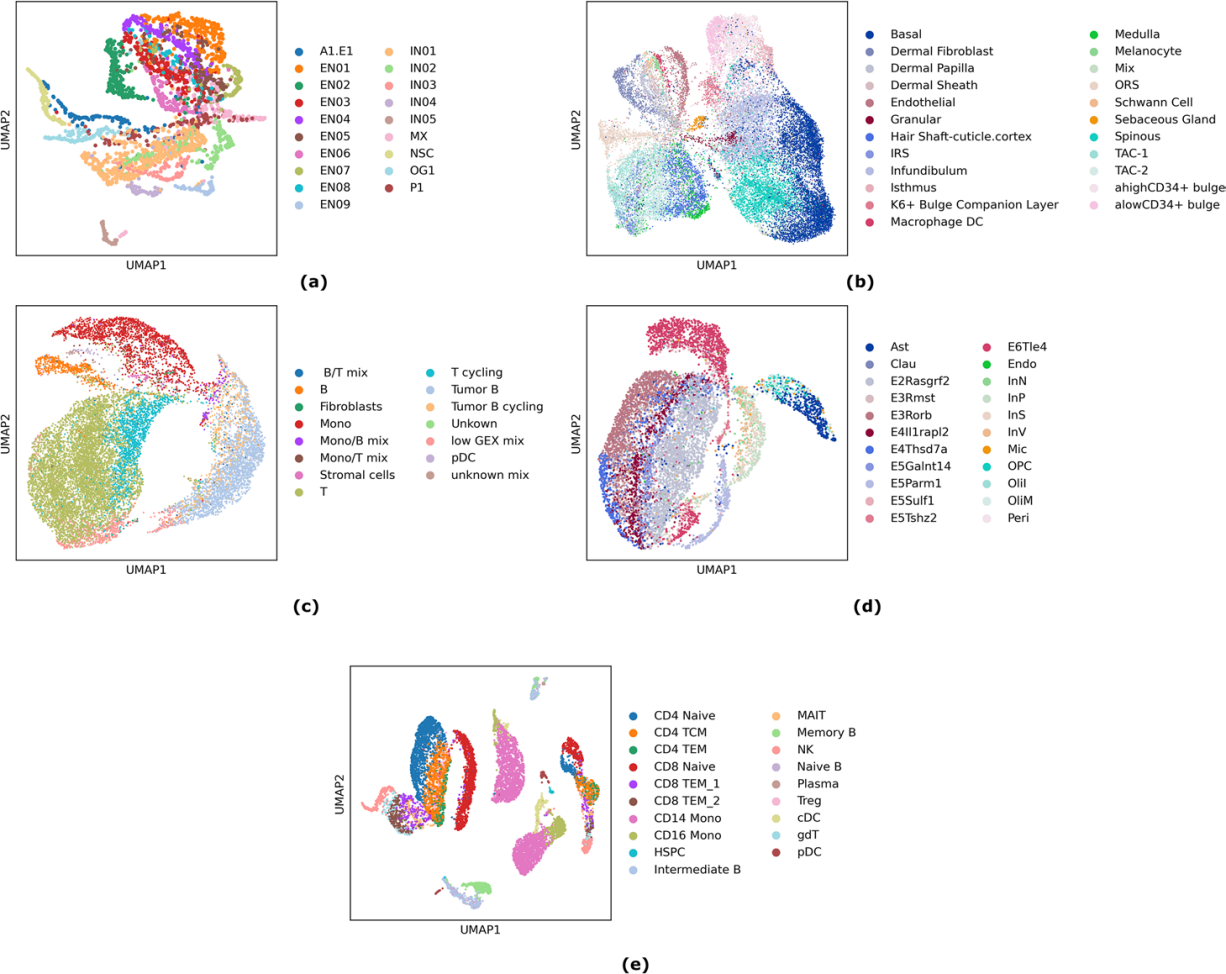
UMAP embedding of SHARE-Topic based on cell-topic distribution($\theta ^c$). **a** SHARE-seq mouse brain data set embedding of 2781 cells from topic space of dimension 30. **b** SHARE-seq mouse skin data set embedding of 27,782 cells from topic space of dimension 60. **c** B-cell lymphoma data set embedding of 14,566 cells from topic space of dimension 45. **d** SNARE-seq mouse cortex data set of 9161 cells embedded in 50 dimensions. **e** 10x Genomics human PBMC10k of 9631 cells embedded in 45 dimensions

**Table 2:** Table showing the accuracy of *K*-NN classifiers trained on the latent representation of different methods to predict cell types in the five datasets. The KNN classifier is trained on 50% of the cells and tested on the rest with k=10. The standard deviation is computed by training 5 KNN-classifiers on randomly chosen cells for each experiment

	Mouse brain	Mouse skin	B-lymphoma	Pbmc10k	Mouse cortex
MOFA+	0.479 $\pm 10^{-2}$	0.379 $\pm 2\times 10^{-2}$	0.813 $\pm 3\times 10^{-3}$	0.802 $\pm 5\times 10^{-3}$	0.66 $\pm 5\times 10^{-3}$
scGlue	0.803 $\pm 10^{-2}$	0.834 $\pm 2\times 10^{-3}$	0.894 $\pm 2\times 10^{-3}$	0.895 $\pm 3\times 10^{-3}$	0.853 $\pm 4\times 10^{-3}$
Seurat (PCA, LSI)	0.854 $\pm 8\times 10^{-3}$	0.887 $\pm 2\times 10^{-3}$	0.904$\pm 2\times 10^{-3}$	0.896 $\pm 5\times 10^{-3}$	0.863 $\pm 4\times 10^{-3}$
**SHARE-Topic**	0.830 $\pm 10^{-2}$	0.754 $\pm 3\times 10^{-3}$	0.871 $\pm 10^{-3}$	0.880 $\pm 2\times 10^{-3}$	0.756 $\pm 7\times 10^{-3}$

Figure 3 shows the results of this exercise on the data sets we consider. The panels show a UMAP reduction to two dimensions of the (posterior mean) topic vectors assigned to each cell, with each dot colored according to the corresponding cell-type annotation. Visually, all plots highlight a good separation between cell types and a biologically plausible organization of the latent space.

Naturally, SHARE-Topic is not the only method capable of obtaining a latent representation from multi-omic data. To quantitatively assess the performance of SHARE-Topic in the context of the state-of-the-art, we performed dimensionality reduction also using three other methods: Multi-Omic Factor Analysis (MOFA+, [10]) a recent adaptation of the MOFA linear dimensionality reduction method to single-cell multi-omic data; Seurat [12], which combines a principal component analysis on transcriptomic data with a preprocessing of chromatin accessibility using latent semantic indexing (itself a technique closely related to topic modeling); and the very recently proposed graph neural-network (GNN) method scGlue [19], which utilizes an AE strategy within a graph-based deep neural network architecture. We used the MOFA+ implementation within the muon platform [29]; due to a technical problem, related to memory usage, we could not run MOFA+ on the whole mouse skin and lymphoma datasets and therefore subsampled that data set retaining approximately only 25k chromatin regions for the ATAC component.

To assess quantitatively the validity of the latent representation discovered by the various methods, we use a *k*-Nearest Neighbor (*k*-NN, using $k=10$) classifier trained on 50% of the cell type annotations, and evaluate its accuracy in predicting the annotations of the remaining 50% of cells. This was repeated over multiple independent splits (100 splits) to achieve a measure of statistical variability.

The results of this assessment are shown in Table 2, and in Additional file 1: Fig. S1 in terms of confusion matrices. Based on this assessment, Seurat performs best across all data sets, with scGlue or SHARE-Topic a close second. In general, all three methods obtain accuracies of over 75% on all data sets. MOFA+ performs at a comparable level with the other methods on the multiome B-lymphoma and PBMC data sets, but its performance on the other data sets is considerably worse than the other methods (but still very significantly better than random).

To benchmark the quality of the data integration performed by SHARE-Topic, we also compared the performance of SHARE-Topic with the reduced models obtained by considering only the transcriptome or the chromatin accessibility data (right and left arms of the graphical model in Fig. 2). The results are given in Additional file 1: Table S1, and the corresponding visualizations are given in Additional file 1: Figs. S2 and S3. The same exercise was also performed for scGlue and Seurat since they automatically provide separate embeddings for RNA and ATAC prior to data integration^1^.

Additional file 1: Table S1 provides a measure of the effectiveness of the various methods in capturing complementary information from the different data sources. Here, we observe a dependence on the underlying multi-omics platform. In the 10X multiome data sets (B-lymphoma and PBMC10k), performance is largely driven by a single modality (RNA and ATAC resp), with the best performance being actually achieved by using a single modality. On the other three data sets, we see that scGlue’s performance, and, to a lesser extent, Seurat’s performance, is largely driven by transcriptomic data, and in fact it is always better on a single source than on two sources. By contrast, when integrating both modalities (scATAC and scRNA), SHARE-Topic in most cases outperforms its RNA-only or accessibility-only versions, indicating that the model is able to effectively integrate both channels of information. We do not know whether the platform dependence of these results is caused by design choices (e.g., depth of sequencing in one modality) or is somewhat linked to the different technology itself.

### Associating SHARE-Topic results to underlying biology

SHARE-Topic’s performance at identifying effective low dimensional representation supports our hypothesis that the degrees of freedom of the system are far fewer than the dimensions of the very high dimensional spaces of genes and regulatory regions. This hypothesis is shared by all dimensionality reduction approaches developed for single-cell multi-omics. SHARE-Topic, due to its transparent probabilistic formulation, offers a natural way to interpret its results, making it suitable as a hypothesis-generating tool.

One simple approach to interpret SHARE-Topic results is to consider topic assignments at the cell level. For example, one may select all cells with the same dominant topic (largest element of the cell-topic assignment vector $\theta _c$) and then check for enrichment of specific cell types among the selected set.

Alternatively, one may leverage the gene by topic matrix, whose entries $\lambda _g^t$ provide expected expression levels of a gene in a certain topic, to obtain a molecular interpretation of the SHARE-Topic latent space in terms of biological processes associated with each topic. To do so, we first associate genes to a topic by computing an entropic measure of the distribution of gene expression across topics (Additional file 2: Fig. S4, see the “Methods” section). Intuitively, we seek genes which are highly expressed in one (or few) topics, and nearly silent in the others; such genes will have a very low entropy, indicating a distribution across topics which is far from uniform (Additional file 2: Fig. S4). By pre-filtering genes based on low entropy levels, we can then associate each gene to the top topic in terms of corresponding expression. This procedure enables us to associate a set of genes to each topic, which can then be queried for enrichment using tools such as clusterProfiler [30, 31] or GSEApy package [32].

**Fig. 4:**
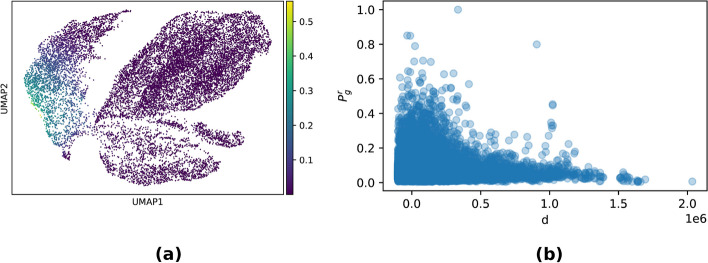
**a** Umap embedding of the B-lymphoma dataset showing the enrichment of topic 13 across cells. Topic 13 is relatively highly enriched in the tumor B cells. This can be an indication that topic 13 captures biological processes specific to B-lymphoma. **b** SHARE-Topic score Prg IN B-lymphoma dataset for gene-region pairs at a distance d of the region from the starting site of the gene (GSS). The regions are selected such that they are on window 105 from the gene. The SHARE-Topic score captures distance dependence. The score decays when going far from the GSS

An example of these analyses is provided in Fig. 4a for the B-lymphoma data set. Here, a particular topic (topic 13 in our run of the algorithm) was strongly concentrated within a particular cell type, tumor B-cells. Considering genes significantly associated with this topic, a number of enriched gene functions appear, primarily but not solely connected with B-cell physiology and tumor biology. A table summarizing the principal functions associated with this topic is provided in Table 3. A similar analysis is carried out for topic 27 in the brain data set, which is strongly enriched in oligodendrocite cells, see Additional file 3: Fig. S6 and Table S1.

**Table 3:** Table showing GO terms of topic 13 enriched in the tumor B cells. The GO terms are obtained using GSEApy package that uses Enrichr to compute the with Fisher’s exact test and adjusted *p*-value with Benjamini-Hochberg method

GO term	Adjusted *P*-value	Odd ratio
DNA damage response	4 $\times 10^{-32}$	3.7
Chromatin remodeling	6.69 $\times 10^{-24}$	4.40
DNA repair	2.42 $\times 10^{-19}$	3.16
Regulation Of DNA repair	1.31 $\times 10^{-11}$	3.84
Regulation Of Cell Cycle	1.10 $\times 10^{-8}$	2.10
Regulation of type I interferon production	3.97 $\times 10^{-7}$	4.86
B cell receptor signaling pathway	1.8 $\times 10^{-5}$	4.69
Response to ionizing radiation	7 $\times 10^{-4}$	2.60
T Cell receptor signaling pathway	8 $\times 10^{-4}$	2.28
B cell homeostasis	0.01	13.23

### SHARE-Topic uncovers regulatory events

One of the most attractive features of sc multi-omic data is the opportunity to identify cell-specific gene-region associations. To do this, we define a score for every pair of genes and chromatin regions (within a certain neighborhood) which quantifies the joint probability of high expression for the gene and of opening for the region. The score is obtained by multiplying a (normalized) expression rate for the gene ($\lambda$ in the notation of Table 1) by the (normalized) open chromatin rate for each region in a pre-defined neighborhood of $10^5$ bp. Region annotations are obtained using the SCREEN database [33]. See the “Methods” section for full mathematical details of the definition of the score. The choice of a very large window of $10^5$bp is designed to capture both distal and proximal regulatory relationships.

In order to validate the proposed score, we first turn to a simulation study. We simulate gene/ region pairs using SCRaPL [20], a recently proposed generative probabilistic model of sc multiomics data that allows to pre-specify the correlation levels between different molecular features. We then correlate the SHARE-Topic score with the relevant prior means in SCRaPL (see the “Methods” section). Figures S10a, b, c, and d show the resulting scatterplots, highlighting a very good recovery of the ground truth parameters despite the differences between the models. We also tested this procedure on Seurat, which also allows the computation of a gene/region association score through Pearson correlation. In this particular set of simulations, Signac [34] did not perform as strongly (see Additional file 4: Fig. S9e and f), possibly due to the difficulties in estimating correlation coefficients from highly noisy data.

As a second, more indirect validation of the biological plausibility of the proposed score, we considered how the score depended on the genomic distance between the gene and the putative regulator. For this analysis, we extended the window around the gene to 2Mb; Fig. 4b shows, on the B-lymphoma data set, that the SHARE-Topic score rapidly decreases after a few hundreds Kb, consistent with the biological intuition that distal regulation over very long genomic distances may be less common. Similar results are shown for the other data sets in Additional file 3: Fig. S8. This empirical decay is remarkable, because the SHARE-Topic model does not in any form encode a notion of genomic distance, so that the distance dependence of the score purely emerges from the data itself.

### SHARE-Topic elucidates the regulation of FOXP1 in B-cell lymphoma

As a biologically relevant example of the use of SHARE-Topic, we turn to the lymphoma multiome data set, studying the regulatory architecture of one of the major regulators. We focus on topic 13; this topic is primarily associated with B-cell tumor cells, and presents a strong enrichment of the cytokine production pathway, indicating a probable involvement in the inflammatory response.

**Fig. 5:**
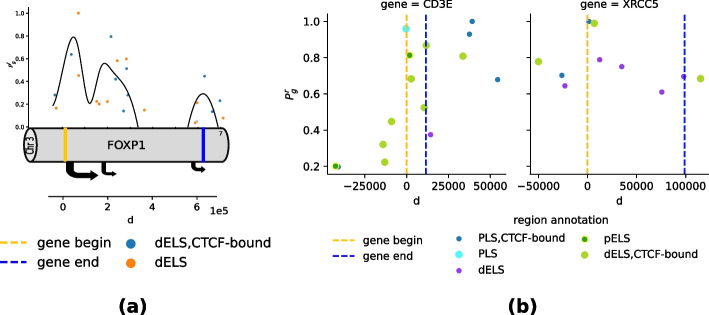
**a** Analysis of the activity of the enhancer in gene FOXP1. According to the SCREEN database, the regions are intersected to distal enhancer-like sites and sometimes also CTCF-bound sites. The SHARE-Topic score is scattered on the open chromatin regions (annotated as enhancers) from the B-lymphoma dataset. The enhancer regions shown are located within a window of $10^5$ within and around FOXP1. The curve is fitted by taking the average of the SHARE-Topic score on intervals of length $10^3$ nucleotide. According to the SHARE-Topic score, the enhancers at the starting site of FOXP1 are shown to have a higher contribution to the gene activity. **b** SHARE-Topic score for regions at distance 105 from two genes (CD35, XRCC5) in the B-lymphoma dataset. The regions are annotated using the SCREEN database as promoter-like sites (PLS), CTCF-bound sites, and proximal/distal enhancer-like sites (p/dELS)

Among the prominent genes associated with this topic, we focus on the master regulator FOXP1, an essential gene in development which has been associated with several cancers, including lymphoma [35]. FOXP1 is a long gene (approximately 600Kb) with a complex transcriptional architecture, expressing several isoform; excess abundance of a short isoform has been reported to be a marker for lymphoma [36]. Brown et al. [36] also observed the presence of two predicted internal regulatory regions just before the start of the short isoform. In our data set, we find several enhancer regions within the gene body, broadly clustered in three positions; Fig. 5a shows the relative importance of the regulatory elements located in or near the gene. We notice a clear dominance of the promoter-proximal regions, but also a substantial support for joint activity in the gene body region. Similar analyses of other genes in the B-lymphoma data set are shown in Fig. 5b and for other data sets in Additional file 3: Fig. S7. While these are purely correlative observations at this level, they provide further examples of the type of non-trivial testable predictions that can be produced by SHARE-Topic.

## Conclusions

Single-cell multi-omic technologies open up unprecedented opportunities to explore the molecular landscape of living cells. Most existing deep-learning based methods have focused on representing the variation in these data sets at the cell level, developing tools which focus on highlighting the diversity across cell populations, but often at the cost of hiding in algorithmic complexity the molecular mechanisms which give rise to this diversity.

With SHARE-Topic, we propose a Bayesian hierarchical model with transparent probabilistic semantics for the analysis of joint expression and chromatin accessibility data. SHARE-Topic provides a low-dimensional representation of multi-omic data by embedding cells in a topic space. We show on a number data sets that SHARE-Topic embeddings are highly accurate at recapitulating cell diversity and effectively integrate both channels of information. Moreover, the simplicity of the model enables a straightforward interpretation of the obtained embeddings in terms of biological processes and permits non-trivial gene-level insights on the interactions of chromatin accessibility and gene expression in single cells.

Topic models have already been employed in a variety of single-cell analyses, ranging from scATAC-seq visualization to determining cellular crosstalk [21, 37]. Since the submission of this paper, we have become aware of a paper using embedded topic models to analyze sc-multiomic data [38], bearing witness to the vitality of this field of research.

SHARE-Topic can also be extended to build more complex data models which will inevitably be needed as single-cell multi-omics technologies become more widely used in biomedicine. Two directions are certainly foremost: first, several other multi-omic technologies such as CITE-seq or scNMT-seq [7, 8] are gaining increasing attention, even though not as widely available. Extension of SHARE-Topic to such technologies is in principle trivial, although the development and implementation of bespoke noise models would be required prior to deployment. Secondly, the increasing availability of commercial kits for sc multiomics will rapidly lead to a proliferation of translational applications in studies with complex experimental designs. This will require the development of models which can dissect variability arising from multiple donors and multiple (possibly related) conditions, creating new challenges for method development. The flexible Bayesian architecture of SHARE-Topic will certainly provide a solid starting points for future models which can address these tasks.

## Methods

### Data filtering

We filtered cells in the skin and mouse brain data sets. In each cell type, cells with a total number of genes read lower than 5–10% or higher than 90% percentile are considered an outlier and discarded in our study. For the three remaining data sets, we kept all the cells. Also, we kept the genes that are expressed in over 5% of the cells.

Since the chromatin accessibility data is binary and highly sparse, filtering the regions is decided according to the average number of region reads across cells. For all data sets except the mouse brain, we retain regions present in at least $\sim$1% of all cells. For the mouse brain data set, which has lower coverage in scATAC-seq, the threshold is 10 fold less ($\sim$0.1%).

### SHARE-Topic implementation

SHARE-Topic is designed to derive from the multiome dataset (transcriptome and chromatin accessibility) a regulatory topic space of dimension *T* (number of topics). The implementation is based on latent Dirichlet allocation [22] and extends to include multiple inputs to infer interaction between inputs in the reduced dimension topic space. SHARE-Topic infers:

1. $\theta ^c = \left( \theta ^c_1,\theta ^c_2,...,\theta ^c_T\right)$: probability distribution of topics in a cell c. $\theta ^c_t$ represents the contribution (importance) of a topic *t* to a cell *c*.
2. $\lambda _g^t$: Poisson rate for gene reads in a topic, i.e., the average number of expected reads when the gene is contributing to a topic *t*. The lambdas are considered independent in our model across topics and genes.
3. $\phi ^t= \left( \phi ^t_1,....,\phi ^t_R\right)$: probability distribution of regions in a topic. $\phi ^t_r$ is the likelihood to observe a region *r* in a topic *t*.

SHARE-Topic is implemented using a Gibbs sampler and the update equations are derived based on the SHARE-Topic graphical model shown in Fig. 2. The latent variables are initialized from predefined priors:

$$\theta ^{c,0} \sim Dir(\alpha ); \lambda _g^{t,0}\sim Gam(\gamma ,\tau ); \phi ^{t,0} \sim Dir(\beta );$$ where:

$\alpha , \beta$: pseudo-count for Dirichlet distribution

$\gamma , \tau$: shape and scale parameters respectively of the gamma distribution

Using the conjugacy property between the priors and likelihood, the Gibbs update equations of the model at the *k*th step are written as follows:

1 $$\begin{aligned}{} & {} P_{g,t}^{c,k} = \frac{\textrm{Poi}\left( n_g^c|\lambda _g^{t,k-1}\right) \theta _t^{c,k-1}}{\sum _{t=1}^{T}\textrm{Poi}\left( n_g^c|\lambda _g^{t,k-1}\right) \theta _t^{c,k-1}} \end{aligned}$$

2 $$\begin{aligned}{} & {} P_{r,t}^{c,k} = \frac{\phi ^{t,k-1}\theta _t^{c,k-1}}{\sum _{t=1}^{T}\phi ^{t,k-1}\theta _t^{c,k-1}}\end{aligned}$$

3 $$\begin{aligned}{} & {} z_g^{c,k}\sim \textrm{Multi}\left( P_{g,1}^{c,k},...,P_{g,t}^{c,k},...,P_{g,T}^{c,k}\right) \end{aligned}$$

4 $$\begin{aligned}{} & {} z_r^{c,k}\sim \textrm{Multi}\left( P_{r,1}^{c,k},...,P_{r,t}^{c,k},...,P_{r,T}^{c,k}\right) \end{aligned}$$

5 $$\begin{aligned}{} & {} \theta ^{c,k} \sim \textrm{Dir}\left( \alpha +N^c\right) ; N^c = \left( N^{c,k}_1,...,N^{c,k}_T\right) \end{aligned}$$

6 $$\begin{aligned}{} & {} \lambda _g^{t,k} \sim \textrm{Gam}\left( \gamma +n_g^{t,k},\frac{\tau }{N_g^{t,k}+\tau }\right) \end{aligned}$$

7 $$\begin{aligned}{} & {} \phi ^{t,k} \sim \textrm{Dir}\left( \beta +N_r^{t,k}\right) \end{aligned}$$

Such that:

- $P_{g,t}^{c,k}$: probability of a gene *g* read in a cell *c* to have membership in a topic *t*,
- $P_{r,t}^{c,k}$: probability of an observed region *r* in a cell *c* to have membership in a topic *t*,
- $z_g^{c,k}$: topic membership of a gene *g* in cell *c*,
- $z_r^{c,k}$: topic membership of a region *r* in cell *c*,
- $n_g^c$: count of a gene *g* in cell *c*,
- $r^c$: observed region *r* in cell *c*,
- $N^{c,k}_t$: total number of regions and genes that have *t*th membership in cell *c *in the *k*th step.
- $n_g^{t,k}$: total number of reads for gene *g* of *t*th membership in the *k*th step,
- $N_g^{t,k}$: total number of gene *g* across cells of *t*th membership in the *k*th step,
- $N_r^{t,k}$: total number of region *r* across cells of *t*th membership in the *k*th step.

The hyperparameters of the model are fixed such that $\alpha =50/T, \beta =0.1, \gamma =1,$ and $\tau =0.5$. We run the MCMC to obtain 3000 samples where 500 samples are used as burn-in. To reduce the effect of correlations, we considered a single sample every 10 samples. The convergence of the MCMC chain is assisted by monitoring the evolution of the likelihood (Additional file 5: Fig. S10).

The outputs of the Gibbs sampler are three matrices: (1) $\theta$ of dimension K$\times$ C$\times$ T, (2) $\lambda$ of dimension K$\times$ T$\times$ G, (3) $\phi$ of dimension K$\times$ T$\times$ R. Here, K, T, C, G, and R are the number of samples, topics, cells, genes, and regions respectively. The latent parameters are estimated using the mean of the samples.

### Choosing number of topics

The number of topics is chosen according to the widely applicable information criterion (WAIC). Using the samples, WAIC of the model is obtained by computing the log-pointwise- predictive-density (lppd) and the variance in log probabilities for each observation (penalty term) [39]:

8 $$\begin{aligned}{} & {} WAIC(n,r;\theta ,\lambda ,\phi )=-2({lppd\ -\ penalty\ term)}\end{aligned}$$

9 $$\begin{aligned}{} & {} {lppd}: \sum \limits _{c,g,r,t} \log \sum \limits _k \dfrac{1}{K}p\left( n_g^c,r^c|\theta _t^{c,k},\lambda _g^{t,k},\phi _r^{t,k}\right) \end{aligned}$$

10 $$\begin{aligned}{} & {} {penalty\ term}: \sum \limits _{c,g,r,t} \textrm{Var}_{\theta _t^c,\lambda _g^t,\phi _r^t} \textrm{log} p\left( n_g^c,r^c|{\theta _t^c,\lambda _g^t,\phi _r^t}\right) \end{aligned}$$

The WAIC is computed for both datasets for different numbers of topics (Additional file 5: Fig. S11). In cases when the WAIC did not exhibit a clear minimum but continued on a very slow descent, a number of topics was chosen at the beginning of the slow descent. Based on these criteria 30, 45, 60, 50, and 45 topics are chosen for brain, B-cell lymphoma, skin, mouse cortex, and PBMC10k datasets, respectively.

### Computational considerations

The diagnostic plots of convergence of MCMC chains are provided in Additional file 5: Fig. S10 (individual chains for all data sets) and Additional file 5: Fig. S12a (across different chains, B-lymphoma data set). The computing times of the model for varying numbers of topics on the B-lymphoma data set are provided in Additional file 5: Fig. S12b. The SHARE-Topic code is written to run on GPUs to gain computational time and speed up the Gibbs sampler computations, especially on big datasets. SHARE-Topic is trained on NVIDIA A100-PCIE-40GB. The time needed to run a single chain scales linearly with the number of topics (Additional file 5: Fig. S12b).

### Associating genes and chromatin regions to topics

Given that topics are intuitively related to biological processes, we expect genes to be strongly non-uniformly distributed across topics, so that in certain topics they are highly transcribed (the expected transcription value $\lambda _g^t$ is high ) while they are relatively less transcribed or absent in other topics ($\lambda _g^t$ is low). The same argument is valid for the accessible chromatin regions, i.e., chromatin regions are active in certain biological processes, thus accessibility($\phi _r^t$) is higher compared to other topics, or less active in others so $\phi _r^t$ is relatively low. To quantify topic specificity we computed the entropy per gene and region across topics. Genes or regions with high entropy are close to the uniform entropy, meaning that they are expressed (or open) at a similar rate across all topics. Additional file 2: Fig. S4 shows that the entropy of all genes (left) and chromatin regions (right) in the B-lymphoma data set are non-uniform. Thus all genes and regions exhibit a degree of topic specificity. A topic, *t*, is assigned to a gene (*g*)/region (*r*) if the $\lambda _g^t/\phi _r^t$ is above the 90th percentile of the $\lambda _g/\phi _r$ distribution across topics. Naturally, a gene might have similar expression rates in different topics if the two topics were largely overlapping. To quantify the degree of independence across topics, we normalized each $\lambda _g$=$\left(\lambda _g^1,...,\lambda _g^T\right)$ and $\phi _r$=$\left(\phi _r^1,...,\phi _r^T\right)$ by subtracting the mean of the vector and dividing with the standard deviation. Then we calculated the dot product between the topic vectors $\left(\lambda _1^t,...,\lambda _G^t;\phi _1^t,...,\phi _R^t\right)$ divided by the norm of the vector. The results for the mouse brain and skin dataset are shown in the Fig. S8a and b, respectively. The resulting heatmaps are dominated by the diagonal, indicating a good level of independence between the topics.

### Annotating topics

After associating a list of genes to each topic, the GO terms enriched per topic are quantified using the GSEApy package [32] (Table 3).

### Inferring regions-genes interactions

In order to quantify the interactions between the genes and the neighboring regions (100kb), we calculated the SHARE-Topic score $P_g^r$:

11 $$\begin{aligned} P_g^r= \frac{1}{C}\sum \limits _c\sum \limits _t{\lambda _g^{*t} \phi _r^{*t} \theta _t^{*c}}\end{aligned}$$

12 $$\begin{aligned} \lambda _g^{*t}=\frac{\lambda _g^{t}}{\sum _{t'}{\lambda _g^{t'}}}\end{aligned}$$

13 $$\begin{aligned} \phi _r^{*t}=\frac{\phi _r^{t}}{\sum _{t'}{\phi _r^{t'}}} \end{aligned}$$

The rationale behind this formula is the following: we expect interacting gene/region pairs to be most highly expressed/ most probably open in the same topics. By taking the dot product, genes/ regions which satisfy this property will yield a high score, while pairs which are independent will have low scores. The normalization step w.r.t. to expression levels is needed to make the association score independent of expression level. The score is normalized by the maximum with respect to the maximum score.

### Benchmarking with synthetic datasets

We generated synthetic datasets using SCRaPL [20]. SCRaPL generates multi-omic data starting from a multivariate Gaussian distribution with mean $\mu ^j$ such that index *j* denotes the gene-region pair and ranges from 1 to J pairs. The vector $\mu ^j$ is composed of two entries $\mu _1^j$ which is gene-specific and $\mu _2^j$ which chromatin region specific; these represent the prior mean expression levels and open chromatin levels. We compare these quantities with inferences from SHARE-Topic of the interaction score, and with the *z*-score used by Seurat to decide about interacting pairs. Because SCRaPL does not have a concept of topics (all pairs are generated independently), we run SHARE-Topic with different numbers of topics on the synthetic data; we also run with different sizes of simulated data in terms of numbers of simulated genes/cells. The scatter plots in Additional file 4: Fig. S9a, b, c, and d show the recovery of the regulation pattern between the ground truth and the SHARE-Topic features specific parameters (genes and regions). We report the Pearson correlations between the parameters of the two models in Additional file 4: Fig. S9. We compared the results with the *z* score computed by Signac [13]; the relevant scatterplots and Pearson correlations are given in Additional file 4: Fig. S9e and f.

### Supplementary information

**Additional file 1.** Quantifying the recovery of cell types using one modality (scATAC-seq or scRNA-seq) at a time.

**Additional file 2.** Assigning topic membership for genes and regions.

**Additional file 3.** Share-topic score and latent variables interpretability on other datasets.

**Additional file 4.** Benchmarking SHARE-Topic performance recovering regions-gene correlations using synthetic datasets.

**Additional file 5.** Assessing MCMC chains convergence and selection of the number of topics.

**Additional file 6.** Review history.

## Data Availability

The datasets used in the paper are publicly available on NCBI [40] (mouse skin and brain) and [41] (mouse cortex). The multiome datasets, B cell lymphoma and Pbmc10k on 10xgenomics website. Regions annotations are obtained from the ENCODE project at https://screen.encodeproject.org/. SHARE-Topic is implemented in Python. The code to recreate all experiments is available on GitHub [42] and archived on Zenodo [43]. All code and data are provided under a GNU General Public License v3.0.

## Footnotes

1 MOFA+ instead learns a joint latent representation so we cannot evaluate it on individual sources without re-running the code independently.
