# Supplementary material for: SHARE-Topic: Bayesian interpretable modeling of single-cell multi-omic data
Source: Genome Biol. 2024 Feb 23;25:55. doi: 10.1186/s13059-024-03180-3 (PMC10885556; doi:10.1186/s13059-024-03180-3)
Supplement: Supplementary file 1 — Additional file 1. Quantifying the recovery of cell types using one modality (scATAC-seq or scRNA-seq) at a time. [file 13059_2024_3180_MOESM1_ESM.pdf]

SHARE-Topic: Bayesian Interpretable Modelling of Single-Cell Multi-Omic Data

Nour El Kazwini[1] **and Guido Sanguinetti**[1]

[1]Theoretical and Scientific Data Science, Scuola Internazionale Superiore di Studi Avanzati, Trieste, Italy

1 Additional file 1

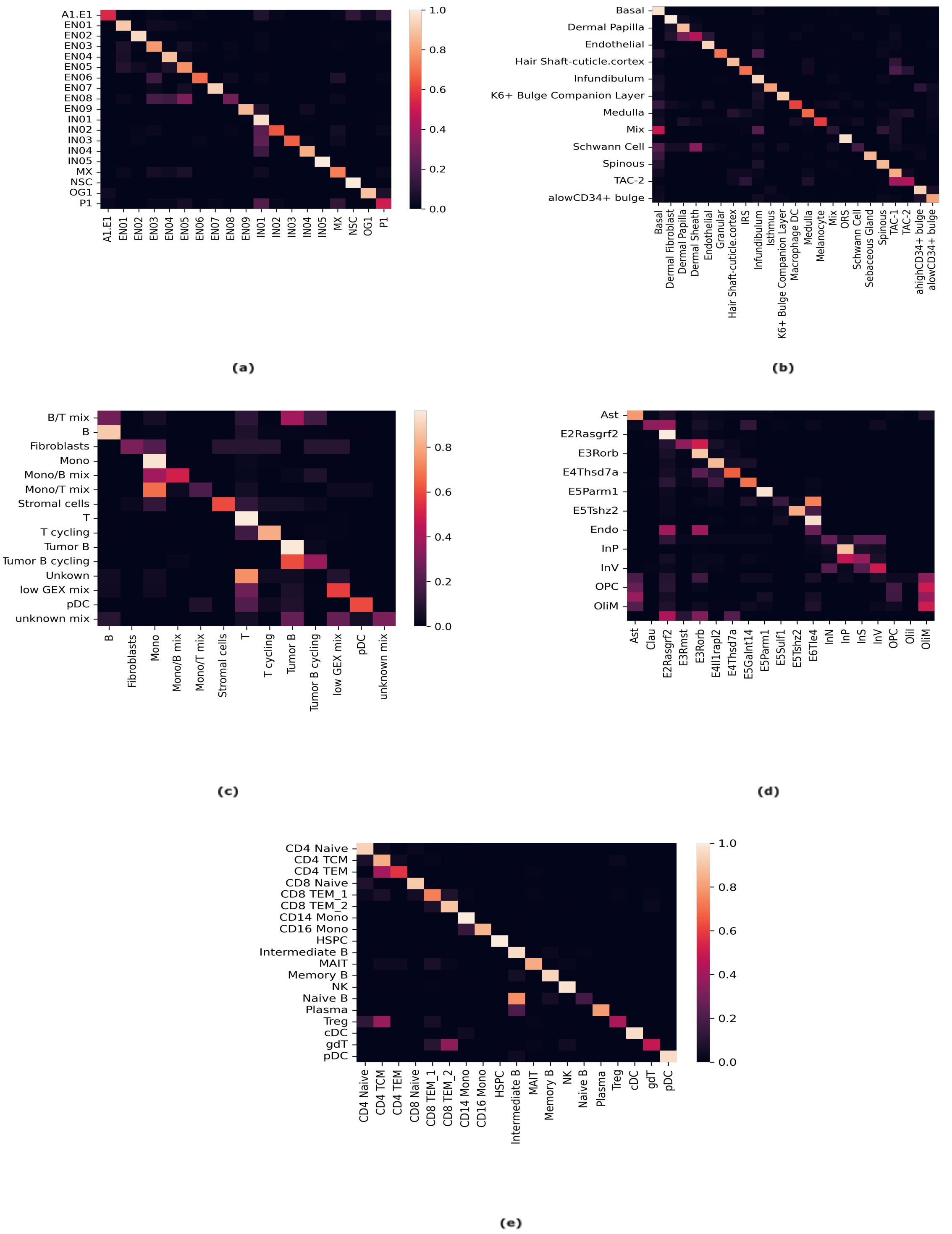

Fig. S1. (a) In the brain data set the classifier has good accuracy in identifying all types except for the case of EN08 it reaches the lowest accuracy 20%. (b) In the skin data set the accuracy is relatively low for two types (ORS, Melanocyte) also 20%. (c) For the B lymphoma data set the KNN do well in identifying strongly presented cell types and fails with the low presented cell population (Mono/B mix). (d) For the mouse cortex data set performs relatively poor in identifying E5SuLf1, OPC, and Olil to the rest of cell types. (e) For Pbmc10k dataset KNN is able to identify most of cell types with high accuracy.

	Mouse brain	Mouse skin	B-lymphoma	Pbmc10k	Mouse cortex
scGlue RNA only	**0.879**$\pm 5\times10^{-3}$	0.838 $\pm 2\times10^{-3}$	**0.911** $\pm 2\times10^{-3}$	0.70$\pm 3\times10^{-2}$	0.866$\pm 4\times10^{-3}$
scGlue ATAC only	0.532 $\pm 10^{-2}$	0.728 $\pm 10^{-3}$	0.781 $\pm 3\times10^{-3}$	0.917$\pm 3\times10^{-3}$	0.749$\pm 4\times10^{-3}$
Seurat (scRNA-seq only)	0.855 $\pm 8\times10^{-3}$	**0.894** $\pm 10^{-3}$	0.907 $\pm 2\times10^{-3}$	0.715$\pm$0.033	**0.868**$\pm 3\times10^{-3}$
Seurat (scATAC-seq only)	0.366 $\pm$0.011	0.683 $\pm 3\times10^{-3}$	0.746 $\pm 4\times10^{-3}$	0.911$\pm 3\times10^{-3}$	0.627$\pm 7\times10^{-3}$
SHARE-Topic RNA only	0.683 $\pm 10^{-2}$	0.703 $\pm 3\times10^{-3}$	0.903 $\pm 10^{-3}$	0.662$\pm 3\times10^{-3}$	0.744$\pm 5\times10^{-3}$
SHARE-Topic ATAC only	0.543 $\pm 10^{-2}$	0.736$\pm 2\times10^{-2}$	0.799 $\pm 10^{-3}$	**0.921**$\pm 3\times10^{-3}$	0.740 $\pm 10^{-3}$

Table S1. Table showing the accuracy of K-NN classifiers trained on the latent representation of the cells to predict cell types in the five datasets. Both latent representations are inferred separately by running scATAC-seq and scRNA-seq disjointly on their relevant part in the method. The KNN classifier is trained on 50% of the cells and tested on the rest with k=10. The standard deviation is computed by training 5 KNN classifiers on randomly chosen cells for each experiment.

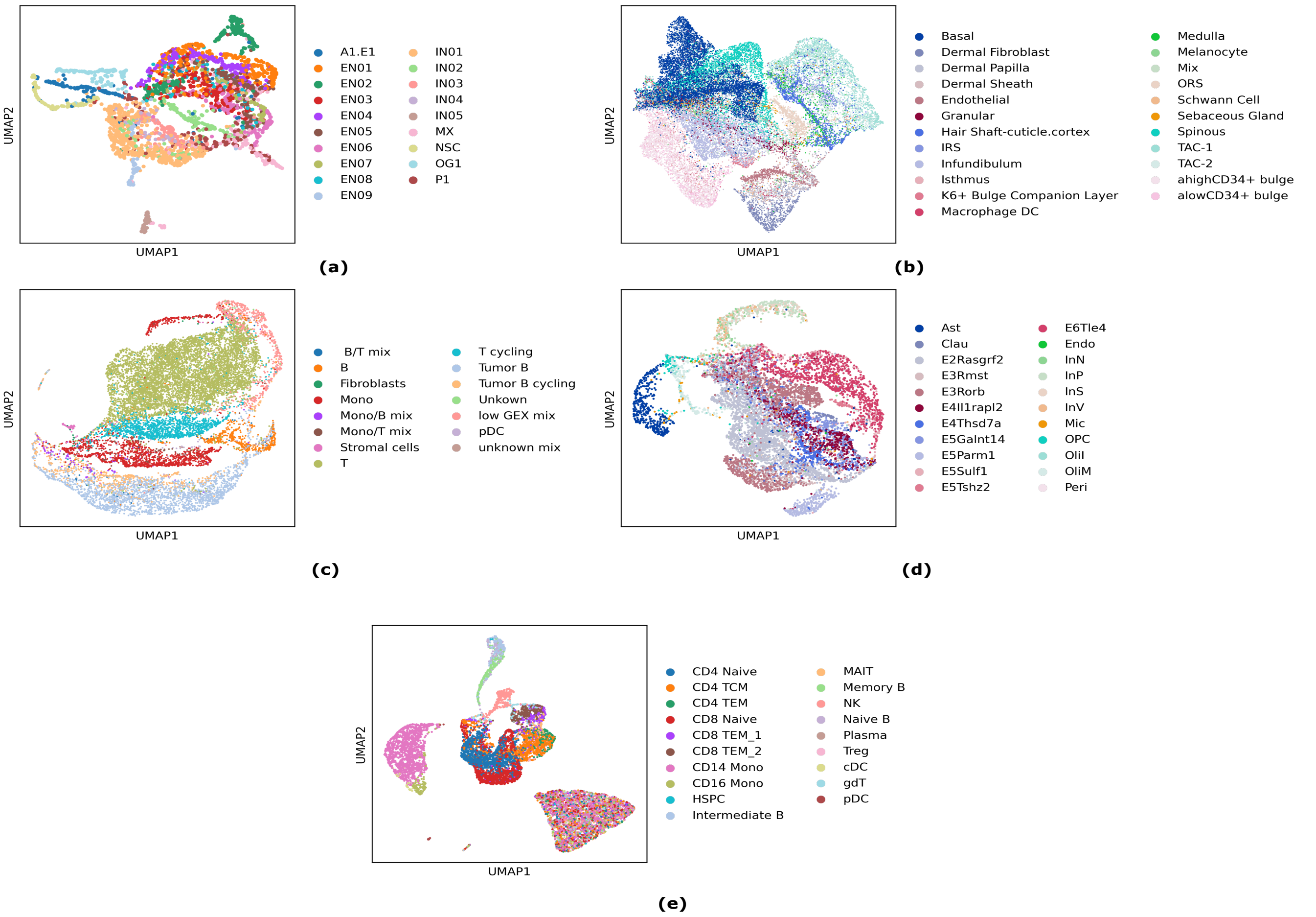

Fig. S2. Umap embeddings of the reduced model considering only transcriptome data. (a) brain dataset embedding of 2781 cells from topic space of dimension 30. (b) skin data set embedding of 27782 cells from topic space of dimension 60. (c) B lymphoma data set embedding 14566 cells from topic space of dimension 45. (d) SNARE-seq mouse data set of 9161 cells embedded in 50 dimensions. (e) 10x Genomics human PBMC10k of 9631 cells embedded in 45 dimensions.

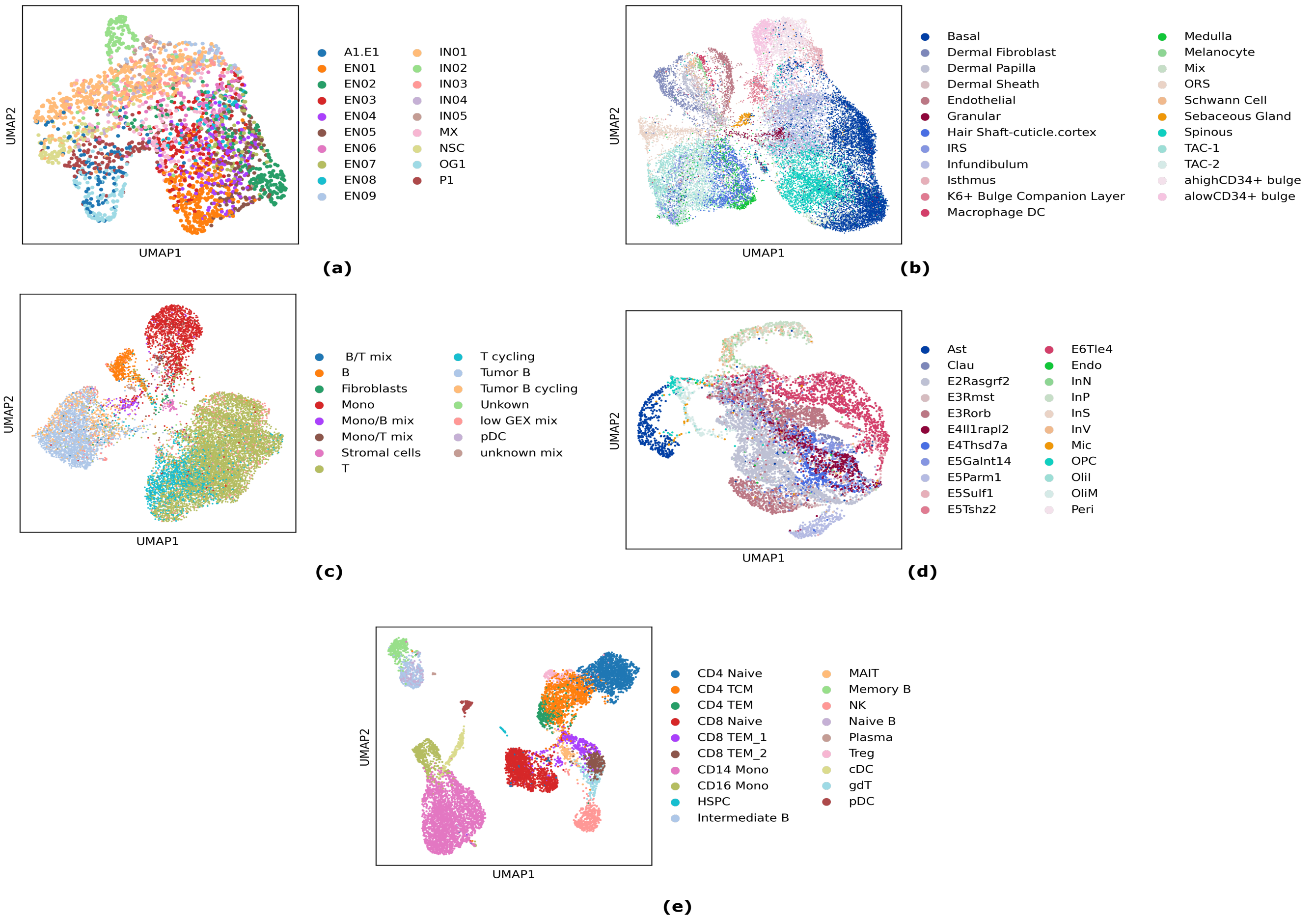

Fig. S3. Umap embeddings of the reduced model considering only chromatin accessibility data. (a) brain data set embedding of 2781 cells from topic space of dimension 30. (b) skin data set embedding of 27782 cells from topic space of dimension 60. (c) B lymphoma data set embedding 14566 cells from topic space of dimension 45. (d) SNARE-seq mouse data set of 9161 cells embedded in 50 dimensions. (e) 10x Genomics human PBMC10k of 9631 cells embedded in 45 dimensions.
