# Supplementary material for: SHARE-Topic: Bayesian interpretable modeling of single-cell multi-omic data
Source: Genome Biol. 2024 Feb 23;25:55. doi: 10.1186/s13059-024-03180-3 (PMC10885556; doi:10.1186/s13059-024-03180-3)
Supplement: Supplementary file 2 — Additional file 2. Assigning topic membership for genes and regions. [file 13059_2024_3180_MOESM2_ESM.pdf]

SHARE-Topic: Bayesian Interpretable Modelling of Single-Cell Multi-Omic Data

Nour El Kazwini[1] **and Guido Sanguinetti**[1]

[1]Theoretical and Scientific Data Science, Scuola Internazionale Superiore di Studi Avanzati, Trieste, Italy

1 Additional file 2

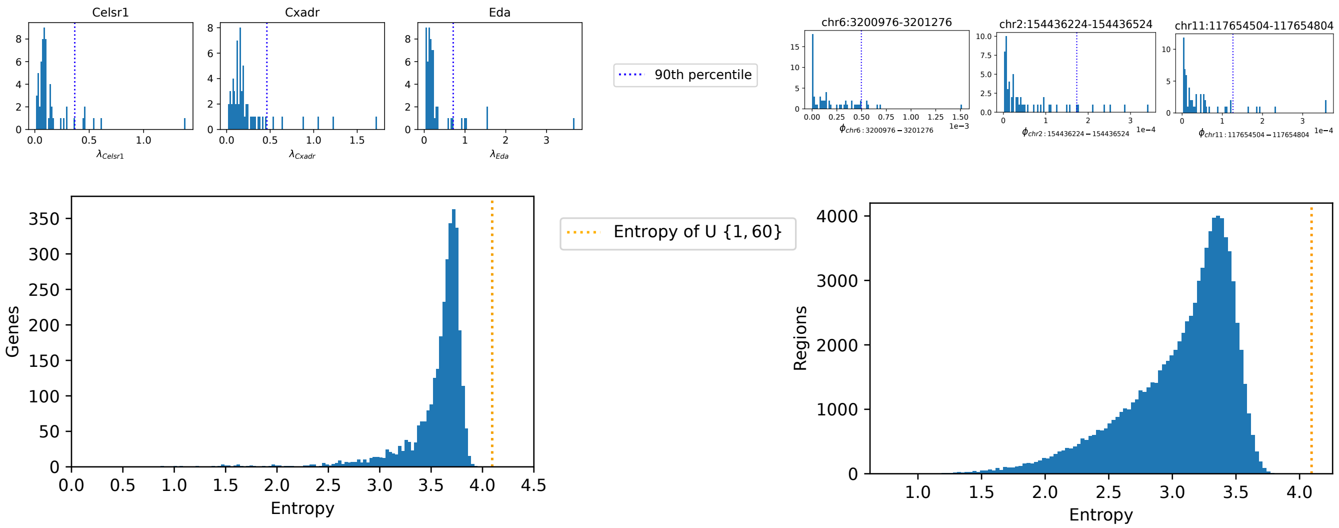

Fig. S4. Entropy distribution of all genes (bottom left) and regions (bottom right) across topics in mouse skin dataset. The number of topics chosen for this dataset using WAIC criterion is 60. The green lines show the maximum entropy obtained from uniform distribution across the 60 topics. All regions and genes have entropy far from being uniform indicating a specificity at the level of topic-membership. The lambdas per gene across topics are shown in the histogram for 3 genes (top left). The phi per region across topics is shown in the histogram for 3 regions (top right). The red line shows the 90th percentile of the read across the topics. The topics with lambdas/phis higher than the 90th percentile of the reads are considered as their values are distinct and higher from the rest of the topics.

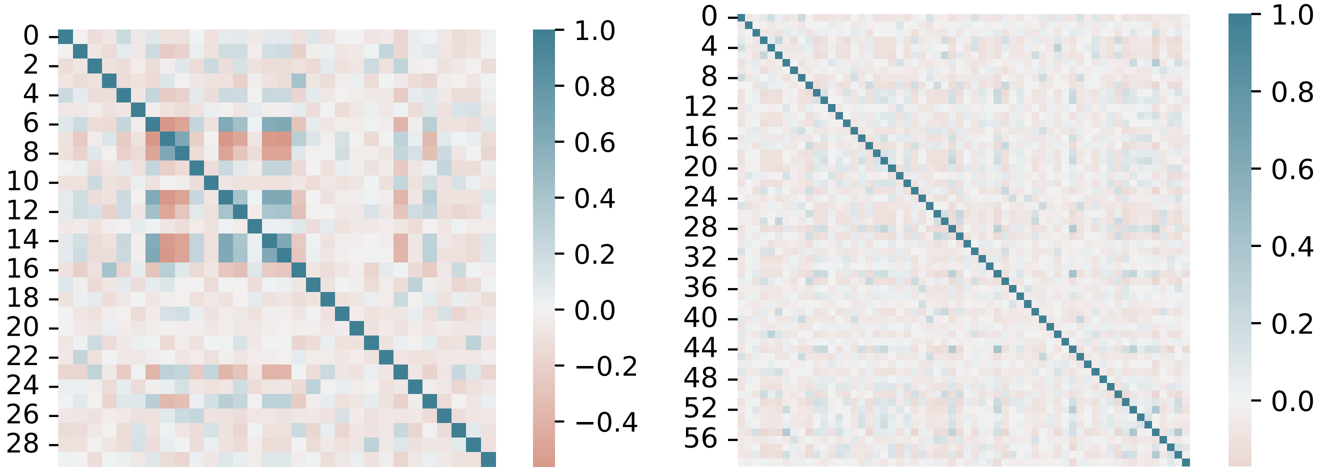

(a) **(b)**

Fig. S5. Heatmap showing the dot products between the topics across lambdas and phi. The lambdas and phis are normalized by subtracting the mean and dividing by the standard deviation. The dot product doesn't show similarity between topics. (a) Heatmap for the mouse brain dataset. 30 topics are chosen using WAIC criteria for this dataset. (b) Heatmap for the mouse skin dataset. 60 topics are chosen using WAIC criteria for this dataset.
