# Supplementary material for: SHARE-Topic: Bayesian interpretable modeling of single-cell multi-omic data
Source: Genome Biol. 2024 Feb 23;25:55. doi: 10.1186/s13059-024-03180-3 (PMC10885556; doi:10.1186/s13059-024-03180-3)
Supplement: Supplementary file 3 — Additional file 3. Share-topic score and latent variables interpretability on other datasets. [file 13059_2024_3180_MOESM3_ESM.pdf]

SHARE-Topic: Bayesian Interpretable Modelling of Single-Cell Multi-Omic Data

Nour El Kazwini[1] **and Guido Sanguinetti**[1]

[1]Theoretical and Scientific Data Science, Scuola Internazionale Superiore di Studi Avanzati, Trieste, Italy

1 Additional file 3

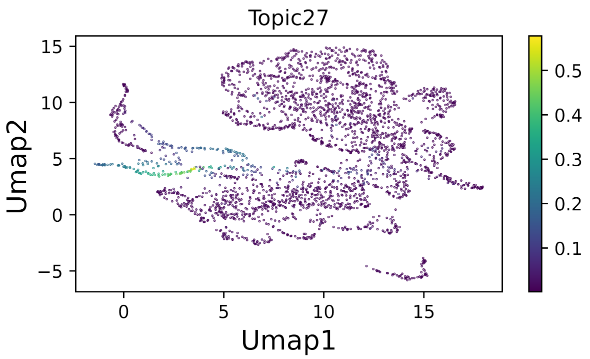

Fig. S6. Umap embedding of the mouse brain dataset showing the enrichment of topic 27 across cells. Topic 13 is relatively highly enriched in Oligodendrocytes (OG). This indicates that topic 27 captures biological processes specific to OG cells.

GO term	Adjusted P-value	Odd ratio
Regulation Of Amyloid-Beta Formation	0.10	10.25
Negative Regulation Of Amyloid Precursor Protein Catabolic Process	0.10	9.29
Oligodendrocyte Differentiation	0.10	13.89
Regulation Of Endocytosis	0.10	6.10

Table S2. Table showing GO terms of topic 27 enriched in the Oligodendrocytes. The GO terms are obtained using GSEApy package that uses Enrichr to compute the with Fisher's exact test and adjusted p-value with Benjamini-Hochberg method. The GO terms are relevant with OG.

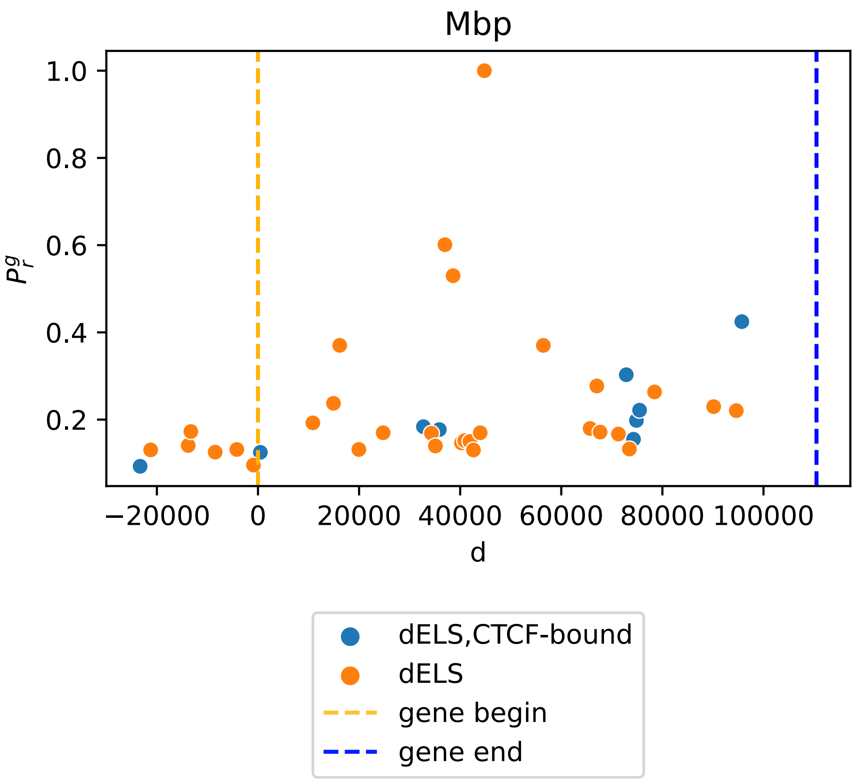

Fig. S7. Analysis of the activity of the enhancer in gene Mbp. According to the SCREEN database, the regions are intersected to distal enhancer-like sites and sometimes also CTCF-bound sites. The SHARE-Topic score is scattered on the open chromatin regions (annotated as enhancers) from the mouse brain dataset. The enhancer regions shown are located within a window of 10^5 within and around Mbp. According to the SHARE-Topic score, the enhancers in the middle of the Mbp are shown to have a higher contribution to the gene activity.

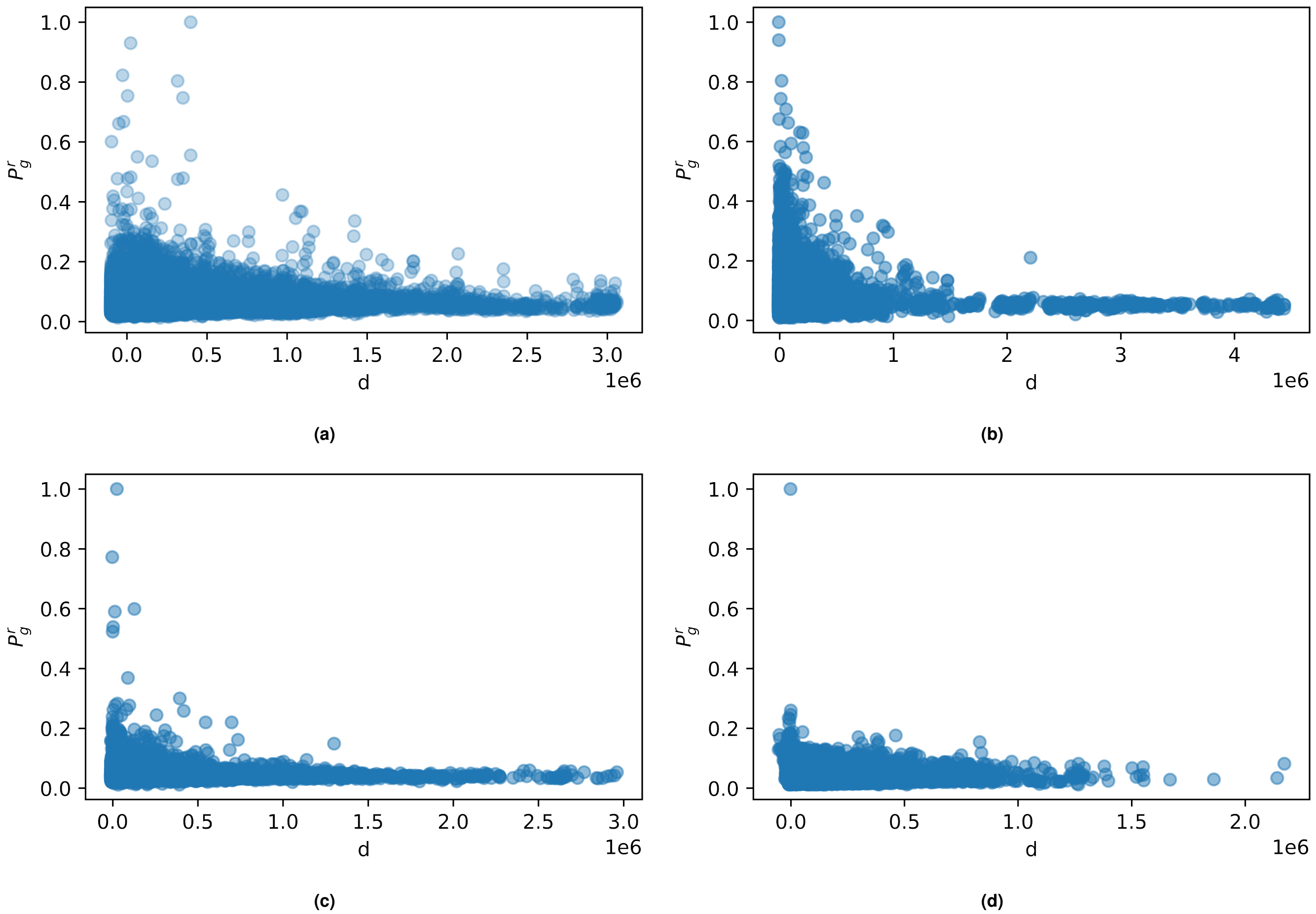

Fig. S8. SHARE-Topic score P_g^r for gene-region pairs at a distance d of the region from the starting site of the gene (GSS). The regions are selected such that they are on window 10^5 from the gene. The SHARE-Topic score capture distance dependence. This is observed across the datasets as the score decaying when going far from the GSS. (a)mouse brain, (b) mouse skin,(c) mouse cortex, and (d) Pbmc10k data sets.
