# Supplementary material for: SHARE-Topic: Bayesian interpretable modeling of single-cell multi-omic data
Source: Genome Biol. 2024 Feb 23;25:55. doi: 10.1186/s13059-024-03180-3 (PMC10885556; doi:10.1186/s13059-024-03180-3)
Supplement: Supplementary file 4 — Additional file 4. Benchmarking SHARE-Topic performance recovering regions-gene correlations using synthetic datasets. [file 13059_2024_3180_MOESM4_ESM.pdf]

SHARE-Topic: Bayesian Interpretable Modelling of Single-Cell Multi-Omic Data

Nour El Kazwini[1] **and Guido Sanguinetti**[1]

[1]Theoretical and Scientific Data Science, Scuola Internazionale Superiore di Studi Avanzati, Trieste, Italy

1 Additional file 4

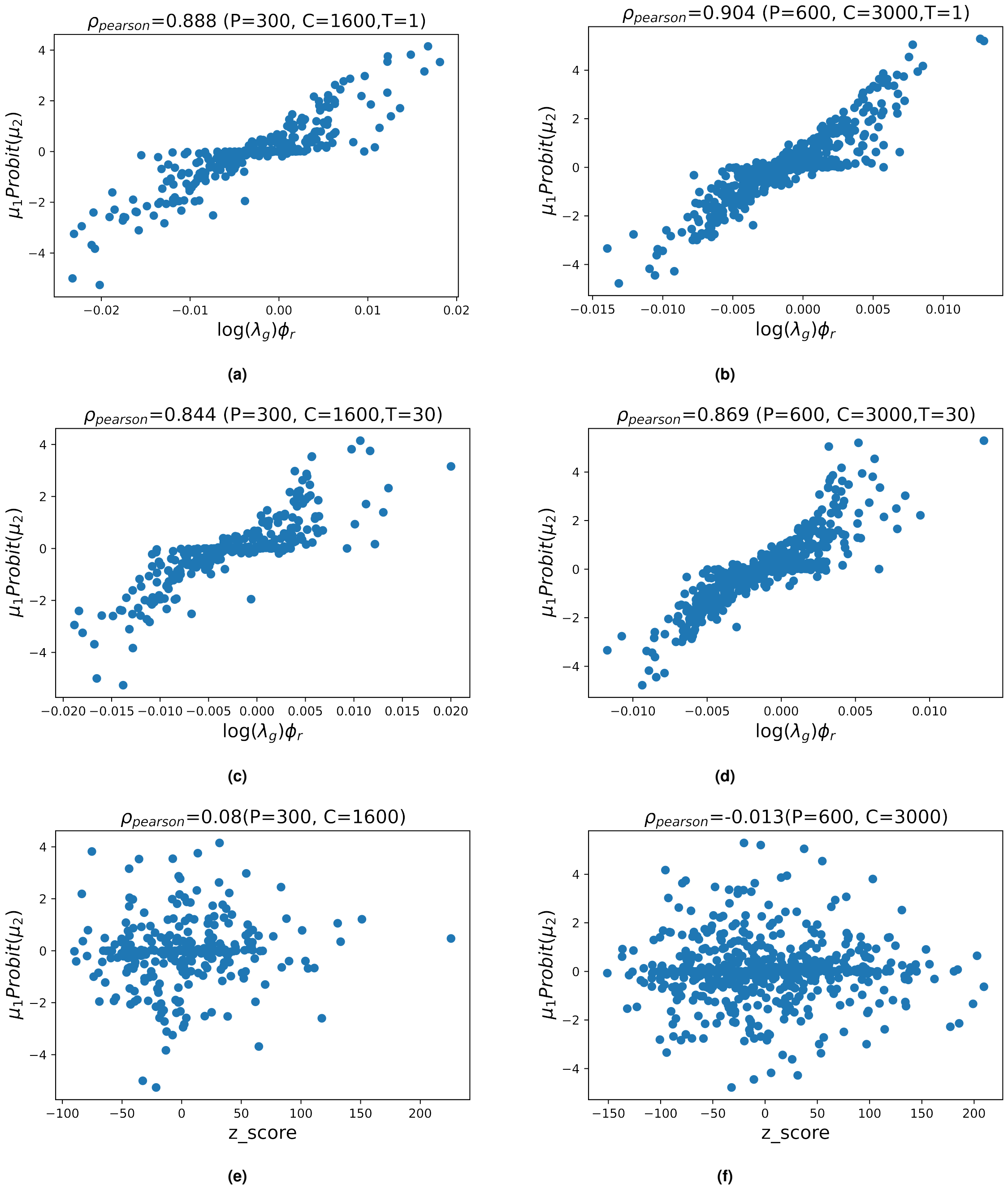

Fig. S9. Figures showing the performance of SHARE-Topic vs Seurat to recover the interaction strength pattern from the data generated using SCRaPL quantified through the pear. In the figures, μ_1 and μ_2 are feature-specific model parameters for the interacting gene and region respectively. The parameters P, C, and T designate the number of region-gene pairs, cells, and topics respectively. (a), (b), (c), (d): Scatter plot for the product region and gene feature parameters versus the feature-specific parameters of SHARE-Topic. Each point corresponds to a pair and the correlation between the two axes is high indicating that SHARE-Topic ability to recover the interaction patterns. We show results for different number of topics: (a) and (b) 1 topic while (c) and (d) 30 topics. SHARE-Topic is robust with a different number of topics. (e) and (f) product μ_1 and probit(μ_2) versus the z score computed using the Signac approach that is based on Pearson correlations. Pearson correlations don't recover the interacting pairs of the SCRaPl's synthetic data.
