# Supplementary material for: SHARE-Topic: Bayesian interpretable modeling of single-cell multi-omic data
Source: Genome Biol. 2024 Feb 23;25:55. doi: 10.1186/s13059-024-03180-3 (PMC10885556; doi:10.1186/s13059-024-03180-3)
Supplement: Supplementary file 5 — Additional file 5. Assessing MCMC chains convergence and selection of the number of topics. [file 13059_2024_3180_MOESM5_ESM.pdf]

SHARE-Topic: Bayesian Interpretable Modelling of Single-Cell Multi-Omic Data

Nour El Kazwini[1] **and Guido Sanguinetti**[1]

[1]Theoretical and Scientific Data Science, Scuola Internazionale Superiore di Studi Avanzati, Trieste, Italy

1 Additional file 5

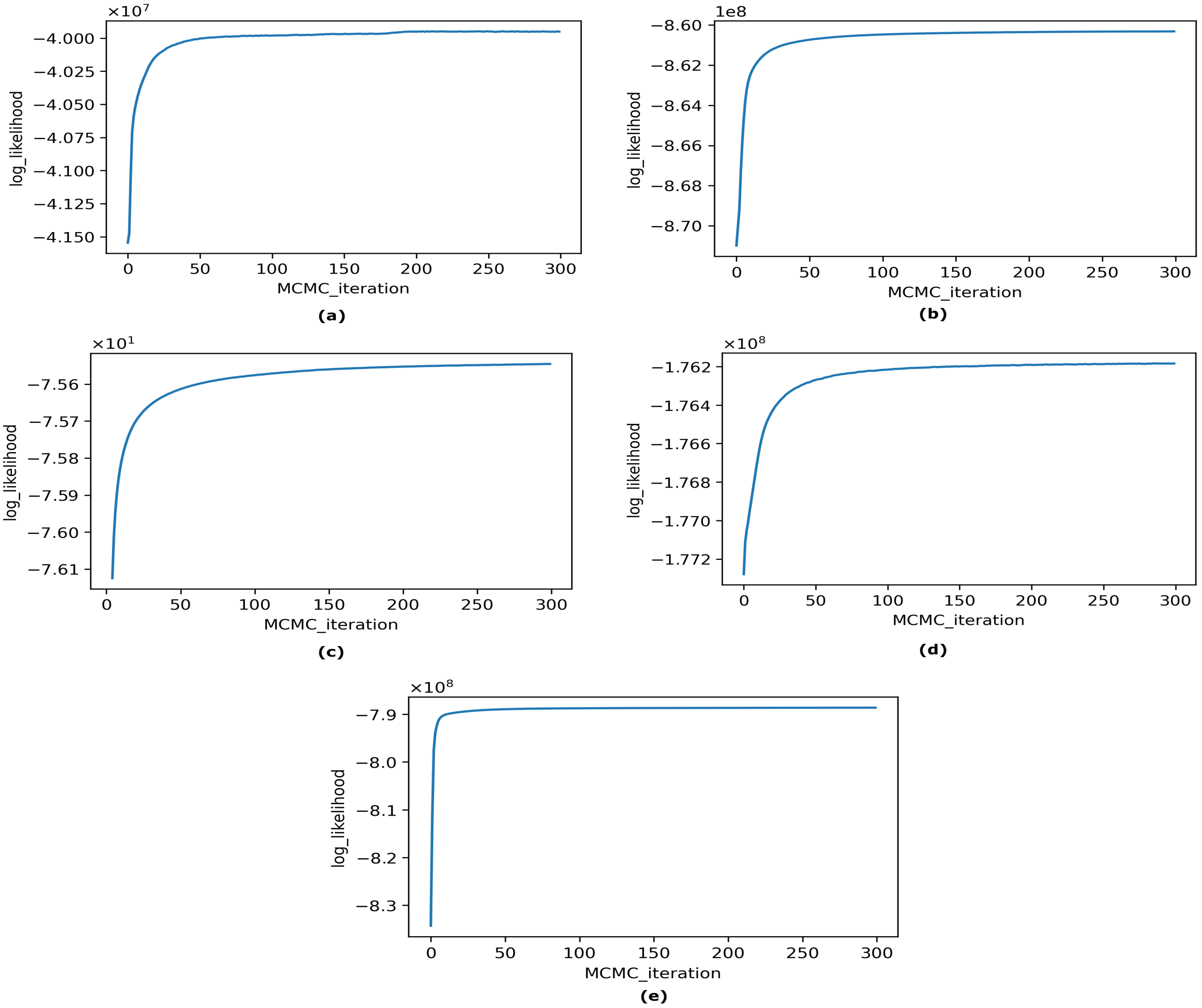

Fig. S10. assessing convergence of the MCMC chains using the log-likelihood for (a)mouse brain, (b) mouse skin,(c) B-cell lymphoma, (d) mouse cortex, and (e) Pbmc10k data sets. The log-likelihood stabilizes after 50 samples(500 samples without thinning).

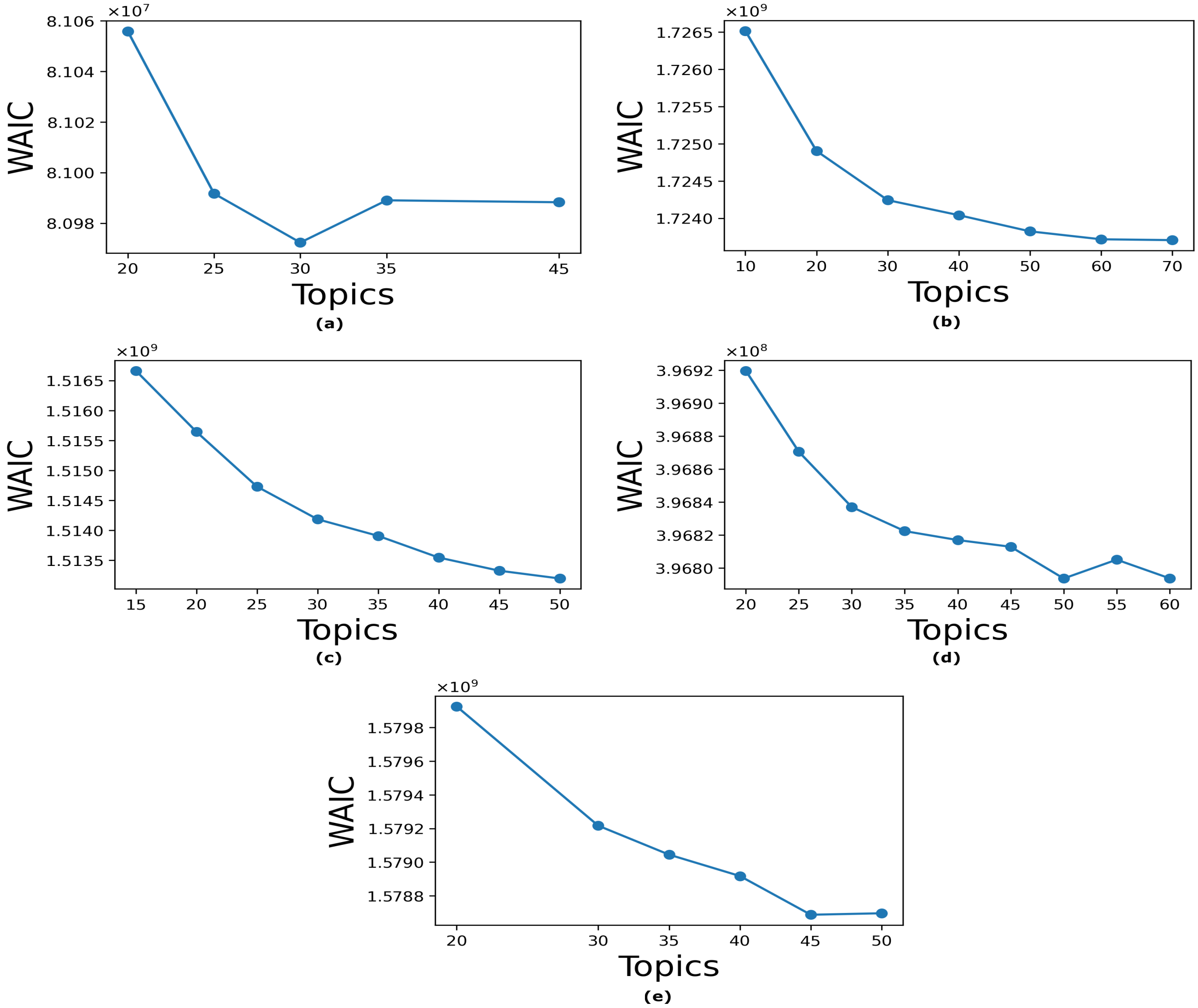

Fig. S11. evolving of WAIC with the number of topics. (a) For the brain data set a minimum is reached at 30 topics. (b) for the skin data set we choose 60 topics. (c) for the B lymphoma data set it is 45. (d) for the mouse cortex data set we choose 50 topics. (e) for the Pbmc10k data set we choose 45 topics. For figures b and c we choose the number of topics such curve become relatively not sensitive when increasing topics.

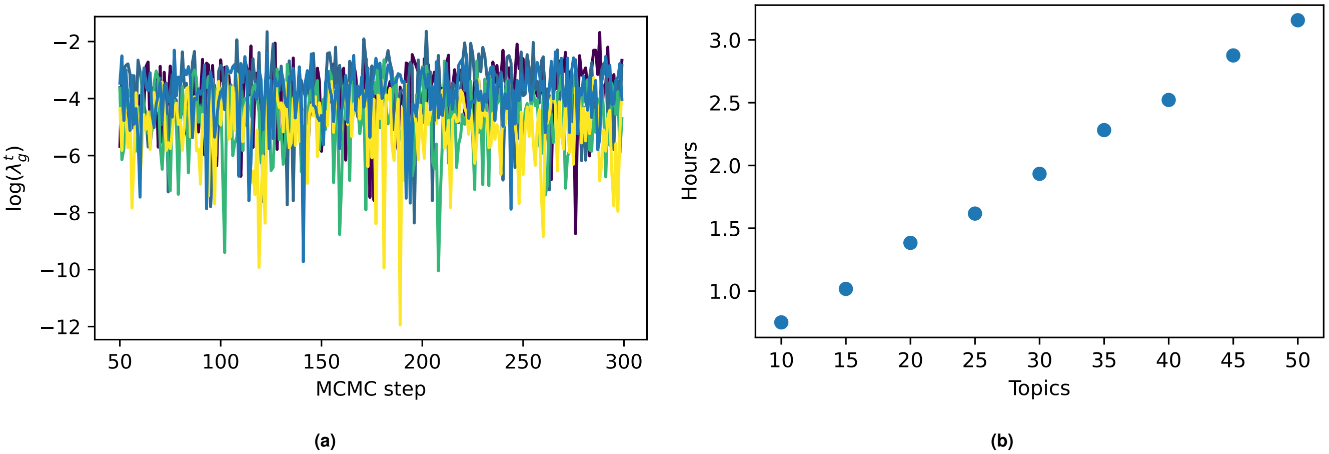

Fig. S12. (a) Four MCMC chains of the Gibbs sampler run on the B-lymphoma dataset. The total number of samples is 3000 such that 9 samples are thrown away between two samples in the figure to mitigate correlations. The chains in the figure are for sampling a lambda parameter (in log scale) of a specific gene and topic. At the end of the sampling we retain 300 samples and discard the first 50 samples according to the likelihood plot, (b) The time cost to run Gibbs sampler of SHARE-Topic when increasing the number of topics for the B-lymphoma dataset. The number of cells, genes, and regions are 14566, 8095, and 94145 respectively. The cost increase is linear with the topic number. GPUs are utilized to run SHARE-Topic to gain speed.
