# Supplementary material for: SHARE-Topic: Bayesian interpretable modeling of single-cell multi-omic data
Source: Genome Biol. 2024 Feb 23;25:55. doi: 10.1186/s13059-024-03180-3 (PMC10885556; doi:10.1186/s13059-024-03180-3)
Supplement: Supplementary file 6 — Additional file 6. Review history. [file 13059_2024_3180_MOESM6_ESM.docx]

Review History

**First round of review**

**Reviewer 1**

**Comments to author:**

This manuscript presents a new method to identify topics from scMulti-omics dataset. The authors demonstrate that the low-dimensional representations obtained by the topic model is comparable to existing methods such as MOFA+ and scGlue, and the new method is able to identify novel gene regulatory pathways related to topics. I have several concerns:

1. From results in Table 2, it seems to me that Share-topic has a consistently worse performance (4 in 5 datasets) than scGlue in identifying low-dimensional representations. So I guess the main novelty of Share-topic will be to identify gene-CRE associations conditional on topics. However, the evaluation of gene-CRE association is severely lacking in the current manuscript. Thus, it is currently not clear what’s the advantage of Share-topic. The authors need to compare Share-topic with other existing tools, for example Seurat/Signac, ArchR, SCENT (https://www.medrxiv.org/content/10.1101/2022.10.27.22281574v1), in simulation studies with gold standard and in real datasets with direct or indirect benchmark of evaluating methods. A potential possibility for simulating data is to use scDesign3 (https://www.nature.com/articles/s41587-023-01772-1). This is both to evaluate if Share-topic can accurately identify gene-CRE associations without generating a lot of false positives, and to show the performance advantage over other existing methods.

2. Related to 1, in addition to numerical comparison, it could be very important to show in real biological examples why we need topic modeling at all. For example, what new biological findings we cannot find without topic modeling, if we use a traditional way of first identifying cell types, and then perform gene-CRE associations within each cell type.

3. Can the model be applied to other types of multi-omics dataset as well, say CITE-seq (gene and protein) and scNMT-seq (gene and DNAm)?

4. It’s not directly clear what the ‘regions’ mean and how were they obtained. Are the ‘regions’ peaks identified by some peak-calling algorithm? Were they downloaded from the original studies?

5. The algorithm uses Gibbs sampler. I guess that will be extremely slow for scMulti-omics datasets with large number of peaks and cells. The authors need to report running time and memory cost to ensure the algorithm can be finished in reasonable time.

6. Association genes and regions with topics seems rather arbitrary. For example, why the top 30% genes/peaks were selected instead of 10%, 20%? Why the top two topics were picked instead of top 1,3,4,5,… A more systematic approach needs to be used to associate genes and regions with topics.

7. Some topics may be highly similar and share the same function. It could be beneficial to allow region-gene pairs across different topics. Currently they have to be with the same topic.

8. Instead of only considering region-gene pairs that are nearby, if considering all possible region-gene pairs regardless of distance, will the region-gene pairs belonging to the same topic more likely to be nearby compared to region-gene pairs belonging to different topics?

9. Surprised to see there is no software implementation (maybe I am missing something). How can the user use the method without a publicly available software?

10. Encode citation is wrong. Check https://www.encodeproject.org/help/citing-encode/

**Reviewer 2**

**Comments to author:**

The authors develop SHARE-Topic, a topic modelling approach for multi-omic single-cell data. The model is an extension of a latent Dirichlet allocation (LDA) model, with inference performed using MCMC (Gibbs sampling). The authors compare to MOFA+ and a recent GNN method (scGlue) in a number of examples for which joint ATAC and expression data are available. The manuscript is generally well-written and the applications and comparisons seem appropriate and fair. I have a few comments that I hope might be useful to help the authors further strengthen their manuscript:

Major points

1. Software availability and more information about its implementation and applicability:

With sincerest apologies if this is just something that I missed, I could not find the implementation of SHARE-Topic. Making this available will be crucial in order for this method to be taken up, so it would be great if information about availability could be included in the manuscript. (Again: please accept my apologies if I have just missed this). It would also be appreciated if some information could be included regarding the implementation (e.g., which programming language, so that I know how difficult/easy it will be for me to install/use), as well as some benchmarking in terms of run times relative to the competing methods that are considered. Also, is SHARE-Topic currently implemented in such a way that an arbitrary number of omics layers could be considered? This was not clear from the description in the manuscript.

2. Use of multiple MCMC chains when performing inference: As far as I can tell (from Fig S1), it looks like a single chain is used in each analysis. It would be great if the authors could include an assessment of variability in inference across multiple MCMC chains for at least one of their examples, and provide some comments and guidance about what to do with the resulting output (particularly if there is disagreement across chains).

Minor points

1. Ordering of some sections: (a) In the literature review part of the manuscript, the authors highlight VAE-based methods, but then in their comparison they compare to MOFA+ and a GNN method. This is slightly jarring for a reader (who might then wonder why the authors have not compared to these VAE methods). I think this would be straightforwardly addressed by including MOFA+ and scGlue up top in their literature review. (b) In Table 1, would it make more sense to have the “Topics” row as the third row in the table, since the symbol “t” is used in some of the other rows?

2. Detail of captions: Particularly for Figs 1 and 2, more detail in the captions would be appreciated. For Fig 1, a bit more explanation of what is going on in the bottom left panel would be great, and for Fig. 2 perhaps just a forward reference to the relevant section where the model and notation are laid out.

3. Possible additional references: I am not an author on any of the following manuscripts, and I will leave it to the authors’ discretion to decide whether or not any of these are relevant, but I wondered if it might be appropriate to cite any of the following manuscripts: (a) Single-cell multi-omic topic embedding reveals cell-type-specific and COVID-19 severity-related immune signatures. Manqi Zhou, Hao Zhang, Zilong Bai, Dylan Mann-Krzisnik, Fei Wang, Yue Li bioRxiv 2023.01.31.526312; doi: https://doi.org/10.1101/2023.01.31.526312 (b) Pancheva A, Wheadon H, Rogers S, Otto TD (2022) Using topic modeling to detect cellular crosstalk in scRNA-seq. PLoS Comput Biol 18(4): e1009975. https://doi.org/10.1371/journal.pcbi.1009975 (c) Gong, B., Zhou, Y. & Purdom, E. Cobolt: integrative analysis of multimodal single-cell sequencing data. Genome Biol 22, 351 (2021). https://doi.org/10.1186/s13059-021-02556-z

4. Choice of number of topics: In the paragraph at the end of the “Choosing number of topics” section, the number of selected topics is only provided for 3 of the datasets analysed. Also, it is not clear from this section how the WAIC is actually used to determine the number of topics. From Fig S2, it is clear that it is not simply the case that the value for which the WAIC is minimal is chosen (since for some of the subfigures the WAIC is continuing to decrease). For clarity, I think this needs a small amount of discussion in the “Choosing number of topics” section.

5. Choice of hyperparameters and thresholds: I think some brief comment on the choice of the hyperparameters (page 8 - why these values?) would be welcome, as would some some comment on some of the other choices (e.g., “the top 30% of genes with the lowest entropy”, “the top two topics with the highest lambdas”, “top 30% of the regions with low entropy”).

6. Typos/grammatical points etc.: (a) WAIC is referred to as both the “Watanabe Information Criterion” and “Widely Applicable Information Criterion”, and I think for consistency just one of these should be used (probably the latter); (b) page 1: “analysis of the resulting latent spaces have shown to outperform…”, “due to the effective impossibility of reliably interpret…”, and “...the joint variability of individual genes regions…” - I think that these may need checking for language/grammar; (c) page 1: transciptome [typo]; (d) page 2: “than utilises” [typo: should be “then”]; (e) page 2: “we couldn’t run MOFA+” [avoid the contraction]; (f) “to which topic each cell partakes” – I think “in which” would be more grammatical here, but there might be a generally better phrasing for what is meant here; (g) page 4 “larget Mouse skin data set”; (h) page 8: “... the SHARE-Topic graphical model shown in 2” – “Figure 2” would be clearer; (i) page 8: “LDA” - abbreviation not introduced/explained; (j) page 8: “To mild the effect of…” –> “To reduce the effect of…”; (k) page 8: Such that K, T, C, and G are the number of samples, …” - I think you have missed “R” from this list; (l) page 9: “... we carried gene ontology to determine…” - missing word(s)?

**Authors Response**

**Point-by-point responses to the reviewers’ comments:**

1 Response to reviewers

Reviewer comments are copied in *italics*, with our replies in normal typeface.

1.1 Reviewer one comments

*This manuscript presents a new method to identify topics from scMulti-omics dataset. The authors demonstrate that the low-dimensional representations obtained by the topic model is comparable to existing methods such as MOFA+ and scGlue, and the new method is able to identify novel gene regulatory pathways related to topics. I have several concerns:*

We thank the reviewer for the positive and constructive attitude to our manuscript.

1. *From results in Table 2, it seems to me that Share-topic has a consistently worse performance (4 in 5 datasets) than scGlue in identifying low-dimensional representations.*

Table 2 was attempting to convey two separate pieces of information: the ability to identify meaningful latent representations (measured by the accuracy of a kNN classifier of cell types) and the ability of a given tool to integrate effectively the two separate sources of information (transcriptomic and chromatin accessibility, measured by comparing the accuracy of the classifier on representations obtained from both data sources against representations from a single data source). We agree that this was confusing and have now split the table into a Table 2 which only reports results on full data sets, and a supplementary table reporting results on individual data sources. Additionally, we have included Seurat to all comparisons, which turns out to be a remarkably strong benchmark. The take away message is that SHARE-topic is competitive with the best methods in obtaining latent representations, and is generally very effective at integrating data sources. We have re-worked the text around Table

2 to better convey this message.

*So I guess the main novelty of Share-topic will be to identify gene-CRE associations conditional on topics. However, the evaluation of gene-CRE association is severely lacking in the current manuscript. Thus, it is currently not clear what’s the advantage of Share-topic. The authors need to compare Share-topic with other existing tools, for example Seurat/Signac, ArchR, SCENT*

*(*[*https://www.medrxiv.org/content/10.1101/2022.10.27.22281574v1),*](https://www.medrxiv.org/content/10.1101/2022.10.27.22281574v1),) *in simulation studies with gold standard and in real datasets with direct or indirect benchmark of evaluating methods. A potential possibility for simulating data is to use scDesign3 (*[*https://www.nature.com/articles/s41587-023-01772-1)*](https://www.nature.com/articles/s41587-023-01772-1))*. This is both to evaluate if Share-topic can accurately identify gene-CRE associations without generating a lot of false positives, and to show the performance advantage over other existing methods.*

Indeed, identifying gene-CRE associations is a major benefit of SHARE-topic. We agree that a comparison on simulated data was needed; we chose Seurat as it is probably the most widely used method, and, due to its linear nature, is also readily amenable to interpretation. As a simulator we considered the suggested tool scDesign3, but, while scDesign does an excellent job at emulating multi-omics data distributions, we were not able to create multi-omics data with a specific correlation structure between transcriptomic and accessibility, which would be needed to obtain ground truth gene-CRE associations. We therefore used (a slightly modified version of) SCRAPL (Maniatis et al, Plos CB 2022) to generate data, generating five independent replicate data sets. Here, SHARE-Topic exhibited a clearly superior performance compared to Seurat; we suspect this is due to the fact that noise strongly degrades the estimates of correlation which are at the heart of the algorithm used by Seurat (an observation already made several times in the past, including in SCRAPL). We have also compared to Seurat on part of one of the real data sets (it should be pointed out that the authors of Seurat themselves acknowledge that their tool is not suited to large scale inference of gene-CRE associations due to the high computational costs). Here the results are less informative, due to the absence of a ground truth, but also point to a higher statistical power of SHARE-topic.

2. *Related to 1, in addition to numerical comparison, it could be very important to show in real biological examples why we need topic modeling at all. For example, what new biological findings we cannot find without topic modeling, if we use a traditional way of first identifying cell types, and then perform gene-CRE associations within each cell type.*

Indeed, this is a valid point; we are not entirely sure whether the reviewer is suggesting here to pseudo-bulk the data by cell type (which may be problematic when the identification of cell types is not straightforward or in the presence of rare/ subtle cell states). If we keep the analysis at the single cell level (which is our intention here), then we believe this point is addressed by the comparison with Seurat. Naturally, we cannot ”prove” that similar results could not also be obtained by developing a different type of model, nevertheless we think that demonstrating the superiority w.r.t. a widely used state of the art highlights that SHARE-topic is also at least a useful and interesting addition to the single-cell multiomics analysis toolkit.

We have extended the discussion to further comment on these issues.

3. *Can the model be applied to other types of multi-omics dataset as well, say CITE-seq (gene and protein) and scNMT-seq (gene and DNAm)?*

This is a good point which we now elaborate upon in the discussion. In principle, the basic architecture is widely applicable, what would need to be adapted however is the type of noise model (for example, one may need a binomial noise model for DNAm). We have focussed on expression and accessibility because it is (at present) the most widely used sc multiomic method, but a more thorough expansion could be the object of further work.

4. *It’s not directly clear what the ‘regions’ mean and how were they obtained. Are the ‘regions’ peaks identified by some peak-calling algorithm? Were they downloaded from the original studies?*

We thank the reviewer for highlighting this point which we forgot to clarify; we obtained already pre-processed data from the original publications, which were indeed obtained by peak-calling (details of these procedures are provided in the original publications). We have now clarified this point.

2. *The algorithm uses Gibbs sampler. I guess that will be extremely slow for scMulti-omics datasets with large number of peaks and cells. The authors need to report running time and memory cost to ensure the algorithm can be finished in reasonable time.*

We now report compute times in Supplementary Figure S6(b); while surely deterministic algorithms might be faster, the compute time is modest and we believe a reasonable price to pay for accurate quantification of uncertainty, which is needed for our probabilistic gene-CRE associations. All experiments were run on a single workstation (details provided in the caption to Figure S6) so memory requirements are standard for a modern computer.

3. *Association genes and regions with topics seems rather arbitrary. For example, why the top 30% genes/peaks were selected instead of 10%, 20%? Why the top two topics were picked instead of top 1,3,4,5,... A more systematic approach needs to be used to associate genes and regions with topics.*

We thank the reviewer for this observation; we have now defined a more systematic approach based on marginalising over topics, which easily enables us to deal with ALL genes/ regions (although of course the results are generally more interesting for high signal genes). We now describe this procedure in Sec Associating genes and chromatin regions to topics.

4. *Some topics may be highly similar and share the same function. It could be beneficial to allow region-gene pairs across different topics. Currently they have to be with the same topic.*

Indeed, that is a very good point which is neatly addressed by our marginalisation approach when deciding how to associate genes and regions. However, empirically it appears that topics are fairly orthogonal at the level of the genes/ regions involved. Suppl. Fig. S7 shows correlations between topics (measured as similarities between rates across genes/ regions) which tend to be fairly low.

5. *Instead of only considering region-gene pairs that are nearby, if considering all possible region-gene pairs regardless of distance, will the region-gene pairs belonging to the same topic more likely to be nearby compared to region-gene pairs belonging to different topics?*

This is an interesting suggestion and indeed if we do that we see a relatively rapid decay (with genomic distance) of our gene-CRE association score, so that there are very few non-negligible scores at distances of more than a few hundred kbs (new Figure 5). This is remarkable, since notions of genomic distance are not enforced anywhere in SHARE-topic, and this biological fact seems to emerge naturally from the data itself.

6. *Surprised to see there is no software implementation (maybe I am missing something). How can the user use the method without publicly available software?*

We apologize for this oversight, we have now made the relevant github repository available at<https://github.com/Nour899/SHARE-Topic/tree/main>

7. *Encode citation is wrong. Check* [*https://www.encodeproject.org/help/citing-encode/*](https://www.encodeproject.org/help/citing-encode/)

Thank you! We have now fixed this.

**2 Reviewer two comments**

*The authors develop SHARE-Topic, a topic modeling approach for multi-omic single-cell data. The model is an extension of a latent Dirichlet allocation (LDA) model, with inference performed using MCMC (Gibbs sampling). The authors compare MOFA+ and a recent GNN method (scGlue) in a number of examples for which joint ATAC and expression data are available. The manuscript is generally well-written and the applications and comparisons seem appropriate and fair. I have a few comments that I hope might be useful to help the authors further strengthen their manuscript:*

We thank the reviewer for the constructive and positive attitude to our work. *Major points*

*1. Software availability and more information about its implementation and applicability: With sincerest apologies, if this is just something that I missed, I could not find the implementation of SHARE-Topic. Making this available will be crucial in order for this method to be taken up, so it would be great if information about availability could be included in the manuscript. (Again: please accept my apologies if I have just missed this). It would also be appreciated if some information could be included regarding the implementation (e.g., which programming language, so that I know how difficult/easy it will be for me to install/use), as well as some benchmarking in terms of run times relative to the competing methods that are considered. Also, is SHARE-Topic currently implemented in such a way that an arbitrary number of omics layers could be considered? This was not clear from the description in the manuscript.*

Thank you very much for highlighting these oversights, which were also picked up by reviewer 1. We have now made the github page accessible and provide implementation details in the Data and Code Availability section. Regarding the inclusion of an arbitrary number of omics layers, this is in principle feasible but practically would need changes to the current implementation. We have now clarified it in the discussion section, where we discussed adaptation to different multi-omics techniques as a topic for further work.

2. *Use of multiple MCMC chains when performing inference: As far as I can tell (from Fig S1), it looks like a single chain is used in each analysis. It would be great if the authors could include an assessment of variability in inference across multiple MCMC chains for at least one of their examples, and provide some comments and guidance about what to do with the resulting output (particularly if there is disagreement across chains).*

Thank you for this observation, we have now performed simulations using multiple chains; an illustration on the B-lymphoma data set is provided in Suppl. Fig. S6(a), showing good mixing of the chains after burn-in.

Minor points

1. *Ordering of some sections: (a) In the literature review part of the manuscript, the authors highlight VAE-based methods, but then in their comparison they compare to MOFA+ and a GNN method. This is slightly jarring for a reader (who might then wonder why the authors have not compared to these VAE methods). I think this would be straightforwardly addressed by including MOFA+ and scGlue up top in their literature review. (b) In Table 1, would it make more sense to have the ”Topics” row as the third row in the table, since the symbol ”t” is used in some of the other rows?*

(a) Thank you for pointing out this oversight on our part; scGlue also involves an autoencoding step within the GNN (although not variational) and in our hands worked better than VAE based methods, which is why we selected it. We have now updated the literature review as suggested. (b) We have swapped the rows in Table 1 accordingly.

2.*Detail of captions: Particularly for Figs 1 and 2, more detail in the captions would be appreciated. For Fig 1, a bit more explanation of what is going on in the bottom left panel would be great, and for Fig. 2 perhaps just a forward reference to the relevant section where the model and notation are laid out.*

Thank you, we have updated the captions to provide more details.

3. *Possible additional references: I am not an author on any of the following manuscripts, and I will leave it to the authors’ discretion to decide whether or not any of these are relevant, but I wondered if it might be appropriate to cite any of the following manuscripts: (a) Single-cell multi-omic topic embedding reveals cell-type-specific and COVID-19 severity-related immune signatures. Manqi Zhou, Hao Zhang, Zilong Bai, Dylan Mann-Krzisnik, Fei Wang, Yue Li bioRxiv 2023.01.31.526312; doi:* [*https://doi.org/10.1101/2023.01.31.526312*](https://doi.org/10.1101/2023.01.31.526312) *(b) Pancheva A, Wheadon H, Rogers S, Otto TD (2022) Using topic modeling to detect cellular crosstalk in scRNA-seq. PLoS Comput Biol 18(4): e1009975.* [*https://doi.org/10.1371/journal.pcbi.1009975*](https://doi.org/10.1371/journal.pcbi.1009975) *(c) Gong, B., Zhou, Y. & Pur-dom, E. Cobolt: integrative analysis of multimodal single-cell sequencing data. Genome Biol 22, 351 (2021).* [*https://doi.org/10.1186/s13059-021-02556-z*](https://doi.org/10.1186/s13059-021-02556-z)

Thank you very much for these additional suggestions, which we now discuss in our literature review. They are indeed relevant, particularly the first one (which was however posted on biorxiv after submission of our manuscript).

4. *Choice of number of topics: In the paragraph at the end of the ”Choosing number of topics” section, the number of selected topics is only provided for 3 of the datasets analysed. Also, it is not clear from this section how the WAIC is actually used to determine the number of topics. From Fig S2, it is clear that it is not simply the case that the value for which the WAIC is minimal is chosen (since for some of the subfigures the WAIC is continuing to decrease). For clarity, I think this needs a small amount of discussion in the ”Choosing number of topics” section.*

We have added the number of topics for the mouse cortex and PBMC data sets, thank you for catching this oversight. Indeed, the reviewer is right; as commonly experienced, information criteria are seldom quite as clear cut as one would hope for on real data. We have added a brief discussion.

3. *Choice of hyperparameters and thresholds: I think some brief comment on the choice of the hyperparameters (page 8 - why these values?) would be welcome, as would some some comment on some of the other choices (e.g., ”the top 30% of genes with the lowest entropy”, ”the top two topics with the highest lambdas”, ”top 30% of the regions with low entropy”).*

Thank you, we have discussed the choice of hyperparameters; now some of the thresholds are no longer used, as we realised that the benefit of introducing the threshold in terms of clearer results was minimal.

3. *Typos/grammatical points etc.: (a) WAIC is referred to as both the ”Watanabe Information Criterion” and ”Widely Applicable Information Criterion”, and I think for consistency just one of these should be used (probably the latter); (b) page 1: ”analysis of the resulting latent spaces have shown to outperform. . . ”, ”due to the effective impossibility of reliably interpret. . . ”, and ”...the joint variability of individual genes regions... ” - I think that these may need checking for language/grammar; (c) page 1: transciptome [typo]; (d) page 2: ”than utilises” [typo: should be ”then”]; (e) page 2: ”we couldn’t run MOFA+” [avoid the contraction]; (f) ”to which topic each cell partakes” - I think ”in which” would be more grammatical here, but there might be a generally better phrasing for what is meant here; (g) page 4 ”larget Mouse skin data set”; (h) page 8: ”... the SHARE-Topic graphical model shown in 2” - ”Figure 2” would be clearer; (i) page 8: ”LDA” - abbreviation not introduced/explained; (j) page 8: ”To mild the effect of... ” -¿ ”To reduce the effect of... ”; (k) page 8: Such that K, T, C, and G are the number of samples, ... ” - I think you have missed ”R” from this list; (l) page 9: ”... we carried gene ontology to determine... ” - missing word(s)?*

Thank you, we have now corrected these small issues.

**Second round of review**

**Reviewer 1**

The review has addressed all my concerns.

**Reviewer 2**

The authors have clearly done a great deal of work toward addressing previous reviewer comments. With one fairly trivial exception (see below), the authors have addressed my points. I also identified some minor typographical/grammatical points that have been introduced in the latest changes, which I also list below.

Minor points:

p2: It appears that the authors may have inadvertently missed the following edit that their response indicated they had intended to address: "(b) In Table 1, would it make more sense to have the ”Topics” row as the third row in the table, since the symbol ”t” is used in some of the other rows?"
p4: "SHARE-topic in integrative modality" - I understand what the authors are saying here, but I am not sure this phrasing completely makes sense
p5/6: "The score is obtained by multiplying a (normalised) expression rate for the gene (λ in the notation of Table 1) times the (normalised) open chromatin rate" - consider "by" instead of "times"
p8/9 "Computational issues" section. I would consider renaming this section as "Computational considerations", simply because "Computational issues" perhaps sounds unnecessarily negative. Moreover, this section might benefit from some additional proofreading, as there are some grammatical and typographic errors, e.g. "singlr", "The SHARE-Topic is trained..." (just "SHARE-Topic", without the definite article, would read better and be consistent with what is written elsewhere), and an abrupt "S7b" at the end of the section.
p9: "Data and code availability" - is there an extra "." at the start of the section?
